# Temporal variation in trophic relationships among three congeneric penguin species breeding in sympatry

**DOI:** 10.1002/ece3.3937

**Published:** 2018-03-05

**Authors:** Arnaud Tarroux, Christian Lydersen, Philip N. Trathan, Kit M. Kovacs

**Affiliations:** ^1^ Norwegian Polar Institute Fram Centre Tromsø Norway; ^2^ Norwegian Institute for Nature Research Fram Centre Tromsø Norway; ^3^ British Antarctic Survey, High Cross Cambridge UK

**Keywords:** Antarctic krill, *Euphausia superba*, interspecific competition, isotopic niche, pygoscelid penguin, stable isotopes

## Abstract

Penguins are a monophyletic group in which many species are found breeding sympatrically, raising questions regarding how these species coexist successfully. Here, the isotopic niche of three sympatric pygoscelid penguin species was investigated at Powell Island, South Orkney Islands, during two breeding seasons (austral summers 2013–2014 and 2015–2016). Measurements of carbon (δ^13^C) and nitrogen (δ^15^N) stable isotope ratios were obtained from blood (adults) or feather (chicks) samples collected from Adélie *Pygoscelis adeliae*, chinstrap *P. antarctica*, and gentoo *P. papua* penguins. Isotopic niche regions (a proxy for the realized trophic niches) were computed to provide estimates of the trophic niche width of the studied species during the breeding season. The isotopic niche regions of adults of all three species were similar, but gentoo chicks had noticeably wider isotopic niches than the chicks of the other two species. Moderate to strong overlap in isotopic niche among species was found during each breeding season and for both age groups, suggesting that the potential for competition for shared food sources was similar during the two study years, although the actual level of competition could not be determined owing to the lack of data on resource abundance. Clear interannual shifts in isotopic niche were seen in all three species, though of lower amplitude for adult chinstrap penguins. These shifts were due to variation in carbon, but not nitrogen, isotopic ratios, which could indicate either a change in isotopic signature of their prey or a switch to an alternative food web. The main conclusions of this study are that (1) there is a partial overlap in the isotopic niches of these three congeneric species and that (2) they responded similarly to changes that likely occurred at the base of their food chain between the 2 years of the study.

## 1 INTRODUCTION

The coexistence of sympatric species and the extent to which their ecological niches overlap are fundamental issues in both theoretical and applied ecology (Silvertown, 2004; Vellend, 2010). Phylogenetically close species, which have less differentiated functional traits (i.e., more overlapping ecological niches, *sensu* Hutchinson, 1957), have traditionally been thought to pose strong competition for one another when they co‐occur (“phylogenetic limiting similarity hypothesis”; Adler, HilleRisLambers, & Levine, 2007; Violle, Nemergut, Pu, & Jiang, 2011). However, this assumption has recently been challenged by theoretical and experimental studies on communities of primary producers showing that species’ phylogenic distances and coexistence can be unrelated (Fritschie, Cardinale, Alexandrou, & Oakley, 2014; Godoy, Kraft, & Levine, 2014). Among high‐trophic‐level consumers, such as seabirds, interspecific competition among closely related and morphologically similar species can be buffered by subtle behavioral adjustments which reduce their ecological overlap, for example, using distinct foraging habitats or resources (Barger, Young, Will, Ito, & Kitaysky, 2016; Robertson et al., 2014). Additionally, the co‐occurrence of several closely related species can result in positive interactions such as the sharing of high‐quality information about where resources are (Anguita & Simeone, 2016; Sridhar et al., 2012). Ecological relationships among closely related species are thus not necessarily purely competitive, which can make understanding them quite challenging.

Penguins are a monophyletic group in which many species are found breeding sympatrically at several sites in the sub‐Antarctic islands (Forcada, Trathan, Reid, Murphy, & Croxall, 2006; Lynch, Fagan, Naveen, Trivelpiece, & Trivelpiece, 2012; Niemandt et al., 2016; Paterson, Wallis, Kennedy, & Gray, 2014; Trathan, Croxall, & Murphy, 1996). The co‐occurrence of these closely related species has long raised questions regarding what degree of competition takes place and how these species successfully coexist (Lynnes, Reid, Croxall, & Trathan, 2002; Trivelpiece & Volkman, 1979; White & Conroy, 1975). This applies particularly to congeneric species such as the three pygoscelids—Adélie *Pygoscelis adeliae*, chinstrap *P. antarctica*, and gentoo *P. papua* penguins, which share similar breeding and foraging ecologies (Hinke et al., 2015; Lynnes et al., 2002; Negrete et al., 2017). In the South Shetland and South Orkney Islands, these three species are found breeding sympatrically at high densities (Levy et al., 2016; Wilson, 2010). It is thought that they achieve coexistence through fine‐tuned ecological segregation mechanisms. Such mechanisms can involve temporal separation of chick‐rearing periods among species (Lynch, Fagan et al., 2012; Trivelpiece, Trivelpiece, & Volkman, 1987), use of spatially distinct foraging habitats in two or three dimensions (Cimino, Moline, Fraser, Patterson‐Fraser, & Oliver, 2016; White & Conroy, 1975; Wilson, 2010), or specialized feeding on different types of prey when sharing the same areas (Hinke et al., 2015; Negrete et al., 2017; Polito et al., 2015). Such closely related species can be expected to reduce the overlap in their ecological niches particularly when resources are limited, as has been observed for instance between Adélie and chinstrap penguins (Lynnes et al., 2002).

Among pygoscelids, the breeding distribution of gentoo penguins reaches much further north than that of the more ice‐tolerant Adélie and chinstrap penguins, but there is an overlap in all three species’ breeding distributions between 54°S and 65°S (Ancel, Beaulieu, & Gilbert, 2013; Black, 2016). Within these overlap areas, breeding times or segregated foraging areas might serve to minimize direct competition. Individual species do show some flexibility. For example, the breeding phenology of gentoo penguins varies widely throughout their breeding range, with later laying dates at more southern latitudes (Black, 2016; Levy et al., 2016). Gentoo penguins also tend to forage closer to shore and deeper in the water column than chinstrap or Adélie penguins (Cimino et al., 2016; Lynnes et al., 2002; Trivelpiece et al., 1987). Despite these differences, and because of their strong reliance on Antarctic krill *Euphausia superba* (hereafter simply referred to as krill) as a food source (Ratcliffe & Trathan, 2011), all three species are listed by the CCAMLR Ecosystem Monitoring Program (CCAMLR 2007) as sentinels of change in critical components of the Southern Ocean food web. Furthermore, penguin species in general are particularly sensitive to, and thus good indicators of, the oceanographic conditions prevailing near their breeding site, as they respond rapidly to fluctuations in resource abundance during the breeding season, through changes in reproductive success within a single breeding season as well as potential short‐term changes in population size (Boersma, 2008; Browne, Lalas, Mattern, & Van Heezik, 2011; Trathan et al., 2015). A better understanding of the mechanisms shaping their respective ecological niches, and how these vary in the context of ecosystem modification through climate change (Miller, Kappes, Trivelpiece, & Trivelpiece, 2010; Polito et al., 2015), would help strengthen their value as biological indicators.

Measurements of stable isotopes of carbon (δ^13^C) and nitrogen (δ^15^N) in consumers’ tissue reflect those of their prey and of the relative proportion of each prey in the consumers’ diet (DeNiro & Epstein, 1978, 1981; Kelly, 2000). Isotopic ratios obtained through a single sampling event can provide dietary information integrated over a period of time that depends on the tissue analyzed as well as the species considered, ranging for instance from a few days in blood plasma to several weeks in red blood cells (Cherel, Connan, Jaeger, & Richard, 2014; Hobson & Clark, 1993). Southern Ocean marine predators such as penguins breed in remote areas and feed at sea, making them challenging to sample regularly for dietary intakes. The use of isotopic measurements that directly integrate their average diet over the past days or weeks can thus prove particularly useful for these species. Recently developed statistical approaches, such as Bayesian niche ellipses (Jackson, Inger, Parnell, & Bearhop, 2010; Swanson et al., 2015), have increased the potential for more refined studies of the trophic niche both at population‐ and individual levels. These methods allow the use of individual isotopic ratios within a given population or group to estimate an n‐dimensional isotopic niche (n depending on the number of isotopes used). The isotopic niche can subsequently be interpreted as a proxy for the realized trophic niche, thereby providing valuable information on the part of a species’ ecological niche that relates to the use of food resources (Newsome, Martínez del Rio, Bearhop, & Phillips, 2007; Yeakel, Bhat, Elliott Smith, & Newsome, 2016). Pygoscelid penguins can feed at various trophic levels, ranging from low‐trophic‐level zooplankton characterized by lower δ^15^N values, such as krill, to squid and fish that are characterized by higher δ^15^N values (Negrete et al., 2017). This gradient in δ^15^N values allows discrimination among individuals/populations feeding mostly on krill vs those feeding mostly on fish/squid species (Juares, Santos, Mennucci, Coria, & Mariano‐Jelicich, 2016; Polito, Lynch, Naveen, & Emslie, 2011).

Herein, the isotopic niche of Adélie, chinstrap, and gentoo penguins breeding at Powell Island, South Orkney Islands, was investigated during two nonconsecutive breeding seasons (2013–2014 and 2015–2016). Our main objectives were firstly to investigate interannual variation/stability in the isotopic niche of each species, and secondly if variation did occur, to determine whether it affected all three species similarly. Specifically, using measurements of carbon and nitrogen stable isotope ratios obtained from tissue samples collected from both adults and chicks, this study (1) quantifies the isotopic niche width and interindividual variation in isotopic ratios in pygoscelids during part of their breeding season, (2) assesses the potential for competition by measuring the interspecific overlap in isotopic niche, and (3) provides an interspecific comparison of the occurrence of an interannual shift in isotopic niche. Based on their phylogenetic relatedness, a strong overlap among the isotopic niches of the three species was expected. Interannual variation in isotopic niche has been shown to occur concurrently in pygoscelid penguins in other areas (Negrete et al., 2017), and it was thus expected that potential changes in isotopic niche would be reflected similarly in all three species.

## 2 MATERIALS AND METHODS

### 2.1 Study site and sample collection

**Figure 1:**
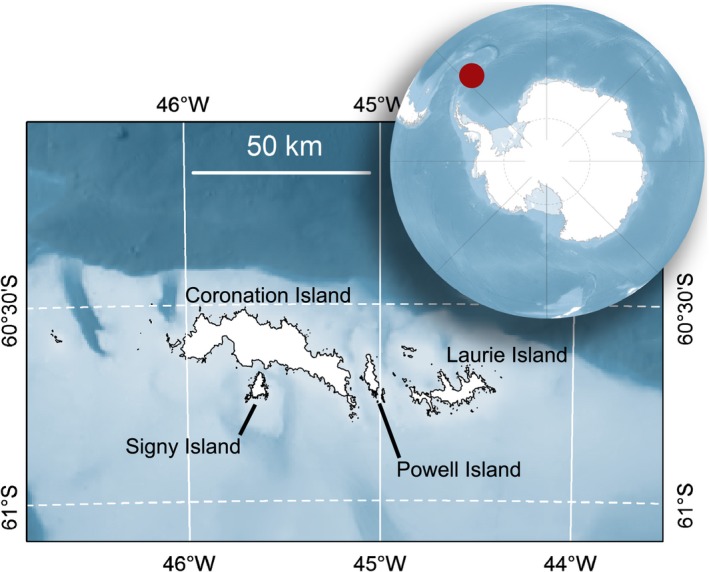
The South Orkney Islands host large breeding populations of Adélie, chinstrap, and gentoo penguins (*Pygoscelis adeliae*,* P. antarctica,* and *P. papua*, respectively). The study was conducted during the austral summers of 2013–2014 and 2015–2016 on Powell Island, where adults and chicks from each species were sampled for isotopic analyses. Continent (Scambos, Haran, Fahnestock, Painter, & Bohlander, 2007) and bathymetric (Dickens et al., 2014) data and are shown only for descriptive purposes

This study focussed on Adélie, chinstrap, and gentoo penguins at breeding colonies on Powell Island (60.73°S, 45.02°W), in the South Orkney Islands (Figure 1), during the breeding seasons of 2013–2014 and 2015–2016 (hereafter 2014 and 2016, respectively). The three species were sampled during each season (Table S1). In the early 1980s, the overall population sizes for Powell Island and the adjacent islets were estimated to be ca. 16,750 Adélie, 28,100 chinstrap, and 8,000 gentoo penguins; more recent estimations are not available for this site (Harris et al., 2015; Poncet & Poncet, 1985).

### 2.2 Sampling for isotopic analyses

Blood sampling of adult penguins took place between (earliest) 22 December and (latest) 9 February in each field season upon their returns from foraging trips. Approximately 1.5 ml of whole blood was collected from the brachial vein into a heparinized tube during each field season, with samples collected during 2016 being centrifuged at ca. 6,700 g during 10 minutes to separate out plasma and red blood cells (RBC). Some plasma samples in 2016 were too small to be processed and analyzed for stable isotopes (Table S1). Each blood component was then stored in 95% ethanol in a separate sterile tube until later analysis. During 2014, it was not possible to centrifuge blood; thus, the entire (whole blood) sample was stored in the same manner. Ideally, different tissues should not be compared directly. However, because whole blood is highly enriched in RBC, one can safely assume that both whole blood and RBC yield similar dietary information through carbon and nitrogen isotopic analyses (Hobson, Schell, Renouf, & Noseworthy, 1996). Therefore, whole blood and RBC samples were pooled into one single group (blood) in all analyses and figures. In the African penguin *Spheniscus demersus*, the half‐life of the ^15^N isotope was estimated to range from 7.6 days in plasma to 14.3 days in red blood cells (Barquete, Strauss, & Ryan, 2013). Blood and plasma isotopic ratios do integrate dietary information over partially overlapping time windows, but herein, it is thus assumed that the measured isotopic ratios integrated dietary information principally over a period of 1–2 weeks for plasma and 2–4 weeks for blood. In order to account for potential confounding factors, the occurrence of an intraseasonal trend in isotopic ratios was examined in the two species for which the temporal coverage of the sampling was long enough within one breeding season to allow testing (chinstrap and gentoo penguins). Using simple linear regressions, only slight temporal trends in isotopic ratios were detected (all absolute trends <0.4‰/month; see details in Figures S1 & S2). Therefore, stationarity of isotopic ratios was assumed throughout each breeding period in all isotopic niche analyses, and the results presented here were assumed to be representative of the average isotopic ratios in the entire month of January. Down and contour feathers (hereafter feathers) were collected from chicks in early February, during both seasons, except for chinstrap penguins, which were sampled only in 2014 (Figure S3). Being naturally built sequentially, down and feather tissues integrate dietary information during the early and late stages of chick growth, respectively (Browne et al., 2011). Both down and feathers were collected simultaneously on each individual with a certain amount of overlap in isotopic ratios of late‐grown down and early‐grown feathers being expected. Subsequent analyses therefore focused only on feather isotopic ratios, although data based on both tissues are presented for comparative purposes.

### 2.3 Sample preparation

In the laboratory, all blood and plasma samples were frozen at −80°C for 24 hr before being freeze‐dried for 48 hr, while down and feather samples were kept dry. Feather samples were washed in an ultrasound bath for 20 min before further processing, to remove dust and other particles. Samples were then powdered using a ball‐mill grinder (blood/plasma) or clipped with fine scissors (down/feather). Some samples were treated to remove lipids (see Section 2.4 below). A small aliquot (target weight 0.4 mg) of each sample was encapsulated into a tin shell before being combusted using a Flash EA 1112 elemental analyzer (Thermo Scientific, Milan, Italy) coupled to a Delta‐V Advantage isotope ratio mass spectrometer via a ConFlo IV interface (Thermo Fisher Scientific, Bremen, Germany). Stable isotope ratios of carbon (δ^13^C) and nitrogen (δ^15^N) are expressed as ‰ of the deviation from isotopic ratios of international standards (Hobson, Piatt, & Pitocchelli, 1994). Acetanilide (Thermo Scientific) and peptone (Sigma‐Aldrich) were used as internal standards and calibrated based on international standards supplied by the International Atomic Energy Agency (IAEA, Vienna, Austria). All mass spectrometry analyses were run at the laboratory of the Littoral Environment and Societies (LIENS) research group at University of La Rochelle, France. The overall measurement precision was evaluated by duplicating a random subset of samples (Jardine & Cunjak, 2005). The mean absolute difference between duplicates was 0.11‰ (95% CI = [0.09; 0.13], n = 102) and 0.10‰ (95% CI = [0.09; 0.12], n = 72), respectively, for δ^13^C and δ^15^N, both measures being well within the analytical precision measures provided by the laboratory (<0.15‰ for both isotopes).

### 2.4 Lipid correction

Lipids in tissues can bias δ^13^C values and dietary interpretation (Logan et al., 2008; Tarroux et al., 2010); high lipid content in animal tissue alters the mass ratio of carbon over nitrogen (C:N ratio), with ratios >4.0 typically indicating significant amounts of lipids (Post et al., 2007). In order to remove surface lipids, down and feather samples were washed using 2:1 chloroform–methanol as solvent and then rinsed in methanol following the method of Jaeger et al. (2013). To develop lipid correction methods suited to this study system, normalization equations were fitted based on a subset of plasma samples for which δ^13^C was measured before and after chemical lipid removal (Wilson, Chanton, Balmer, & Nowacek, 2014). First, lipids were chemically extracted from 46 samples through two successive rinses with 2:1 chloroform–methanol as solvent. Then, normalization equations were estimated by regressing the difference in δ^13^C between lipid‐extracted and bulk plasma samples on the C:N ratio of the bulk samples, using nonlinear least square regression (Ehrich et al., 2010). All δ^13^C values of plasma samples were thus corrected (Table S2) using the normalization equation that best fitted the data (Table S3). All δ^15^N values were left uncorrected as δ^15^N is not affected by lipid content (Yurkowski, Hussey, Semeniuk, Ferguson, & Fisk, 2015). The C:N ratios of whole blood samples were all <3.6, confirming that lipid normalization was not necessary (Table S3).

### 2.5 Statistical analyses

The isotopic data used in the analyses are available from the Norwegian Polar Institute’s data repository https://doi.org/10.21334/npolar.2018.5aadb005. All data were processed and analyzed in R 3.2.5 (R Development Core Team 2017). The normalization equations for carbon isotopic data were determined using the function nls from package stats. Average isotopic ratios were compared among species by means of ANOVAs using function aov from the package stats. The analyses related to the isotopic data and niche computations were conducted using the script from Turner, Collyer, and Krabbenhoft (2010) and the package nicheROVER (Swanson et al., 2015).

For a given year and age class, the relative location of each species within the two‐dimensional isotopic space was compared by computing the Euclidean distance (DIST) among centroids. Additionally, the mean distance to centroid (MDC), an index of trophic diversity within a given group (i.e., dispersion), was computed and compared among species (within year and age class), among years (within species and age class), and among age classes (within species and year; Layman, Arrington, Montana, & Post, 2007). All contrasts were tested statistically against the null hypothesis that difference in DIST or MDC was equal to zero (e.g., for DIST, testing that two species’ centroids are in the same isotopic area), through residual permutation procedures (RPP; Turner et al., 2010), using n = 9,999 permutations.

In order to compare the isotopic niche among years and species, data were plotted using isotopic biplots and niche region (Nr) computed for each year. To calculate credible intervals around the parameter estimates, 10,000 elliptical projections (random ellipses) of Nr were drawn randomly from the posterior distributions. For a given group of individuals, Nr corresponds to the portion of a multidimensional isotopic space (two‐dimensional in this study) where the probability of finding any individual from that group is equal to a given, user‐defined threshold (Swanson et al., 2015). For each year, 95% was used as the threshold defining the global isotopic niche, and the area of the two‐dimensional 95% Nr (Nr_area_) was used as a measure of the trophic niche width. The overlap between the isotopic niches of two species is defined as the probability of an individual drawn randomly from a given species being found in the Nr of the other species. The niche overlap is therefore asymmetrical; overlap between species A and B is not directly equivalent to overlap between species B and A, depending on how evenly each group uses its own niche area (Swanson et al., 2015). Tissue‐specific discrimination factors have not yet been determined in Adélie or chinstrap penguins, and only feathers have been investigated in gentoo penguins (Polito, Abel, Tobias, & Emslie, 2011). Herein, the direct comparison of the isotopic niches of the three species relies on the assumption that the diet–tissue isotopic discrimination factors are similar for all three species. While this assumption is currently unverifiable, results from a study on different penguin species suggest that it is reasonable, when study‐specific discrimination factors cannot be determined, to use an average value for wild fish‐eating birds (Cherel, Hobson, & Hassani, 2005).

Complementary to the niche overlap estimation, the amplitude and direction of temporal isotopic shifts from 2014 to 2016, represented as two‐dimensional vectors in the isotopic space, were compared statistically among species and age class, again using RPP (Turner et al., 2010), with n = 9,999 permutations.

## 3 RESULTS

### 3.1 Isotopic niche width and interindividual variation in isotopic ratios

**Figure 2:**
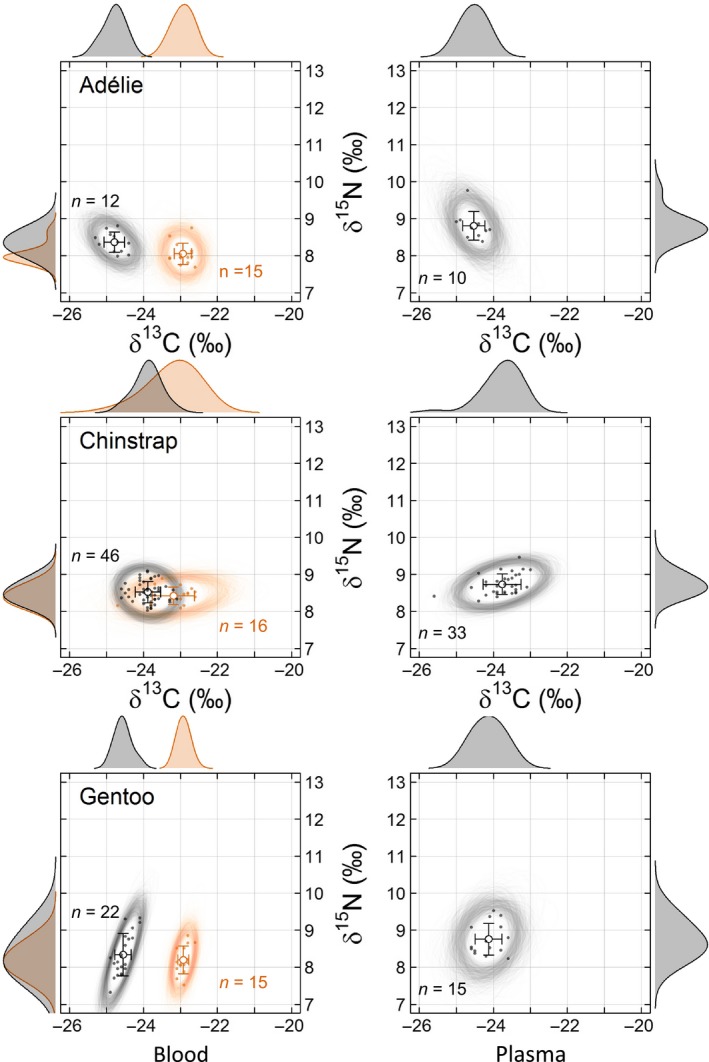
Interannual variation in niche regions areas (Nrarea; represented by 95% random ellipses) based on δ^13^C and δ^15^N of blood and plasma from adult Adélie, chinstrap, and gentoo penguins from Powell Island, South Orkney Islands, in 2014 (orange) and 2016 (black). “Blood” stands for “whole blood” or “red blood cells” (see Section 2 for details). Plasma δ^13^C values are normalized to account for lipid content (see Section 2 for details). Empty circles and error bars show the mean (±*SD*) isotopic ratios. Density curves for each isotope are drawn marginally along the corresponding axis

In adults, the 95% Nr area (hereafter Nr_area_) ranged from 0.9 to 2.3‰^2^ and from 1.8 to 2.7‰^2^, for blood and plasma, respectively (Table 1, Figure 2). Plasma samples were not collected in 2014, and results from 2016 are thus presented for comparative purposes only, but are not discussed further. For blood, the Nr_area_ of gentoo penguins in 2014 was smallest, while that of chinstrap penguins in 2014 was largest (Table 1). There was little variation in isotopic ratios within individual years, species, or tissues, although for gentoo penguins, the variation was three times as high for δ^15^N (*SD* = 0.6‰) compared to δ^13^C (*SD* = 0.2‰) in 2016. For chinstrap penguins, on the other hand, variation along the carbon axis was higher, especially in 2014 (*SD* = 0.6‰). In adults, MDC was generally small (≤0.52; Table 2) and did not vary significantly among species, except in 2014 when chinstrap penguins had higher MDCs than Adélie penguins (difference = 0.20 ‰, *p* = .030; Table 2, 3) and in 2016 when gentoo penguins had higher MDC than Adélie penguins (difference = 0.17 ‰, *p* = .043; Tables 2, 3). There was no interannual variation in MDC detected in adults of any of the species studied (all *p*‐values <.001).

**Table 1:** Mean area (95% credible interval) of the isotopic niche region (Nr) per breeding season, species, age class, and tissue in pygoscelid penguins from Powell Island, South Orkney Islands

	Adults		Chicks	
	Blood	Plasma	Down	Feather
2016				
Adélie	1.2^a^ [0.7; 2.1]	1.8^b^ [1.0; 3.2]	0.9^c^ [0.5; 1.7]	0.9^e^ [0.5; 1.6]
Chinstrap	1.8^a^ [1.3; 2.4]	2.3^b^ [1.7; 3.2]	–	–
Gentoo	1.4^a^ [0.9; 2.0]	2.7^b^ [1.6; 4.5]	4.9^d^ [2.7; 8.8]	4.4^f^ [2.3; 8.1]
2014				
Adélie	1.2^a^ [0.7; 2.0]	–	1.1^c^ [0.7; 1.6]	0.8^e^ [0.5; 1.2]
Chinstrap	2.3^a^ [1.4; 3.8]	–	0.6^c^ [0.4; 0.9]	0.6^e^ [0.4; 0.8]
Gentoo	0.9^a^ [0.5; 1.4]	–	2.0^c,d^ [1.3; 3.0]	3.3^f^ [2.2; 4.9]

“Blood” stands for “whole blood” or “red blood cells” (see Section 2 for details). “Feather” stands for “contour feather”. Superscript letters are identical among Nr areas that are not statistically different from each other, within each tissue (i.e., when their 95% credible intervals intersect). Nr areas are in ‰^2^ and were estimated based on 95% random ellipses (see Section 2 for details).

**Table 2:** Summary of the interspecific differences in mean distance to centroid (MDC, in ‰) between adult and chick pygoscelid penguins from Powell Island, South Orkney Islands

	Adults	Chicks	Empirical *p*‐value
2014			
Adélie	0.31	0.29	.794
Chinstrap	0.51	0.23	**<.001**
Gentoo	0.35	0.65	**.003**
2016			
Adélie	0.35	0.27	.222
Chinstrap	0.40	–	–
Gentoo	0.52	0.79	.340

Results based on isotopic ratios in blood (adults) and feather (chicks). Empirical *p*‐values estimated from permutations procedures (see Section 2 for details) are in bold when significant at α = 0.05.

**Table 3:** Mean Euclidean distance between species’ centroids (DIST, in ‰; upper triangular matrices) and interspecific difference in mean distance to centroid (MDC, in ‰; lower triangular matrices, shaded) based on isotopic ratios in blood of adult pygoscelid penguins from Powell Island, South Orkney Islands

	Adélie	Chinstrap	Gentoo
2014			
Adélie	–	**0.46 (<0.001)**	0.14 (0.506)
Chinstrap	**0.20 (0.030)**	–	**0.36 (0.008)**
Gentoo	0.04 (0.727)	0.17 (0.090)	–
2016			
Adélie	–	**0.93 (<0.001)**	0.24 (0.162)
Chinstrap	0.05 (0.513)	–	**0.71 (<0.001)**
Gentoo	**0.17 (0.043)**	0.11 (0.051)	–

Empirical *p*‐values estimated from permutations procedures are in parentheses (see Section 2 for details) and in bold when significant at α = 0.05.

**Figure 3:**
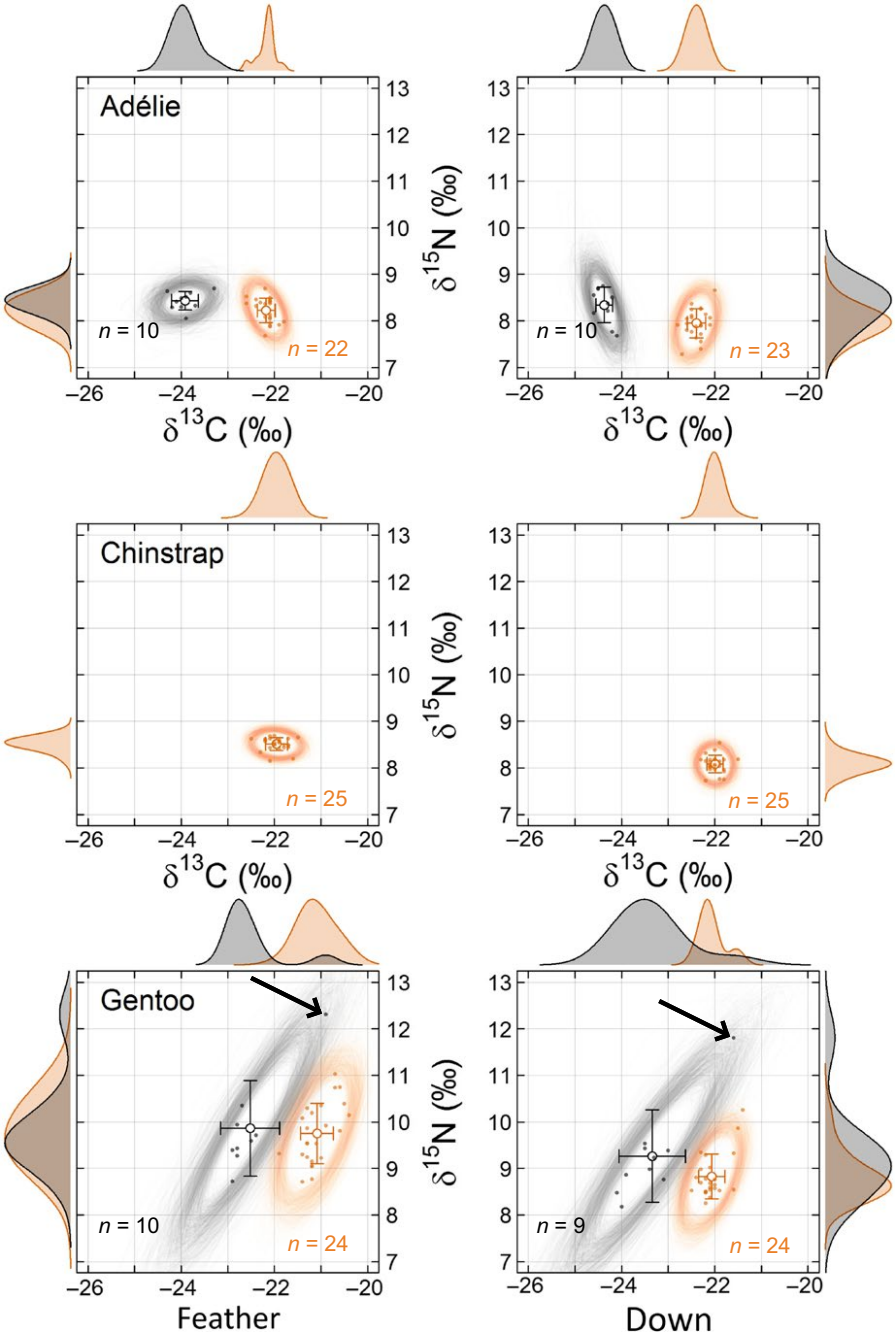
*Interannual variation in niche regions areas (Nr*_*area*_ *; represented by 95% random ellipses) based on δ* ^*13*^ *C and δ* ^*15*^ *N of feather and down from chick Adélie, chinstrap, and gentoo penguins from Powell Island, South Orkney Islands, in 2014 (orange) and 2016 (black). “Feather” stands for “contour feather”. Empty circles and error bars show the mean (± SD) isotopic ratios. Density curves for each isotope are drawn marginally along the corresponding axis. The two arrows indicate the individual points causing an increase in the Nr*_*area*_ *of gentoo penguins in 2016*

In chicks, there were also only slight interspecific differences in isotopic niche width based on either down or feathers (Table 1, Figure 3). For both tissues (feather and down), Nr_area_ values were generally smaller in chicks than in adults (blood and plasma), resulting in more contracted isotopic niches. However, gentoo penguins had the widest isotopic niches. This was particularly accentuated in 2016 due to one individual that was clearly different from other individuals, with notably higher δ^13^C and δ^15^N values (Figure 3). Both the down and feather samples with the highest values corresponded to the same individual and showed a similar difference from the rest of the group, indicating that this was probably not due to an analytical artifact. This was also confirmed by running a duplicate analysis on the downsample. In gentoo chicks, MDC was over twice as large as in Adélie and chinstrap penguins (Tables 2, 4). For comparative purposes, when excluding that individual from the analyses, the Nr_area_ was up to three times narrower. In chicks’ feather, the Nr_area_ then decreased from 4.4‰ (95% credible interval = [2.3; 8.1]) to 1.3‰ (95% credible interval = [0.7; 2.5]). In chicks’ down, when removing that individual from the calculations, the Nr_area_ decreased from 4.9‰ (95% credible interval = [2.7; 8.8]) to 2.3‰ (95% credible interval = [1.2; 4.5]).

**Table 4:** Mean Euclidean distance between species’ centroids (DIST, in ‰; upper triangular matrices and interspecific difference in mean distance to centroid (MDC, in ‰; lower triangular matrices, shaded) based on isotopic ratios in feather of chick pygoscelid penguins from Powell Island, South Orkney Islands

	Adélie	Chinstrap	Gentoo
2014			
Adélie	–	**0.36 (0.003)**	**1.88 (<0.001)**
Chinstrap	0.06 (0.451)	**–**	**1.51 (<0.001)**
Gentoo	**0.36 (<0.001)**	**0.42 (<0.001)**	–
2016			
Adélie	–	–	**2.00 (<0.001**)
Chinstrap	**–**	–	–
Gentoo	**0.52 (0.036)**	–	–

Empirical *p*‐values estimated from permutations procedures are in parentheses (see Section 2 for details) and in bold when significant at α = 0.05.

### 3.2 Isotopic niche overlap among species

Overall, chinstrap penguins were most unique, being situated further apart (i.e., DIST values among species significantly different from zero) from the two other species in the isotopic space in both years; twice as much in 2016 (Figure 2, Table 3). The mean overlap among Nr of adults was large with an average of 48% over both years (Figure 2, Table 5). The isotopic niche of adult gentoo penguins generally had the highest overlap with those of Adélie or chinstrap penguins during both years, ranging from 46% to 84% (Table 5). Contrastingly, adult chinstrap penguins had the lowest overlap with the two other species also during both years, ranging from 12% to 44% (Table 5).

**Table 5:** Mean isotopic niche overlap [95% credible interval] in pygoscelid penguins from Powell Island, South Orkney Islands

	Adélie	Chinstrap	Gentoo
Adults			
2014			
Adélie	–	24.3 [3.7; 58.9]	52.6 [29.8; 76.8]
Chinstrap	12.0 [2.2; 37.3]	–	18.8 [8.7; 32.5]
Gentoo	55.4 [34.8; 80.3]	46.0 [23.7; 68.0]	‐
2016			
Adélie	–	64.0 [33.3; 92.8]	70.4 [46.3; 91.4]
Chinstrap	44.2 [20.9; 71.4]	–	31.9 [18.1; 50.0]
Gentoo	84.0 [61.3; 98.4]	72.5 [47.0; 94.3]	–
Chicks			
2014			
Adélie	–	39.6 [19.0; 64.4]	5.5 [0.0; 50.4]
Chinstrap	52.1 [25.8; 81.0]	–	35.1 [0.5; 88.1]
Gentoo	0.8 [0.0; 3.5]	2.9 [0.4; 7.7]	–
2016			
Adélie	–	–	29.2 [0.4; 86.9]
Gentoo	4.7 [0.2; 15.5]	–	–

Overlap is expressed as the % probability of an individual from species A (rows) to be found in the Nr of species B (columns; see Section 2 for details). Results are based on carbon and nitrogen isotopic ratios measured in blood (adults, Figure 1) and feather (chicks, Figure 2).

In chicks, the average δ^13^C in feathers was significantly different among Adélie and gentoo penguins both in 2014 (ANOVA; *F*_2, 68_ = 105.00, *p* < .001) and 2016 (ANOVA; *F*_1, 17_ = 42.38, *p *< .001). Gentoo penguins had the highest δ^13^C and δ^15^N (Figure 3) in both years. Specifically, in 2014, the average δ^13^C of gentoo penguins was +1.1‰ (95% CI = [0.9; 1.3]) higher than that of Adélie penguins and +0.9‰ (95% CI = [0.7; 1.1]) higher than that of chinstrap penguins (Figure 3). In 2016, gentoo penguins’ average δ^13^C was 1.4‰ (95% CI = [0.9; 1.9]) higher than that of Adélie penguins. Similar differences were found for δ^15^N. In 2014, the average δ^15^N of gentoo penguins was +1.5‰ (95% CI = [1.2; 1.8]) higher than that of Adélie penguins and +1.2‰ (95% CI = [0.9; 1.5]) higher than that of chinstrap penguins (Figure 3). In 2016, the average δ^15^N of gentoo penguins was +1.4‰ (95% CI = [0.7; 2.1]) higher than that of Adélie penguins. Comparable differences were detected when examining isotopic ratios from down tissue, though of lower amplitude (Figure 3). Overall, chicks from all three species occupied different isotopic spaces in both years, with gentoo penguin's chicks being situated furthest apart (Figure 3, Table 4). As a result, the Nr of chicks generally showed less overlap (range: 0.8%–52.1%; Table 5) among the three species than that of the adults (Figure 2). The Nr of gentoo penguin's chicks overlapped those of Adélie or chinstrap penguins by <5% (Table 5). However, the Nr of Adélie and chinstrap penguins overlapped each other quite considerably, up to 52.1% (Table 5).

### 3.3 Interannual variation in isotopic niche

**Figure 4:**
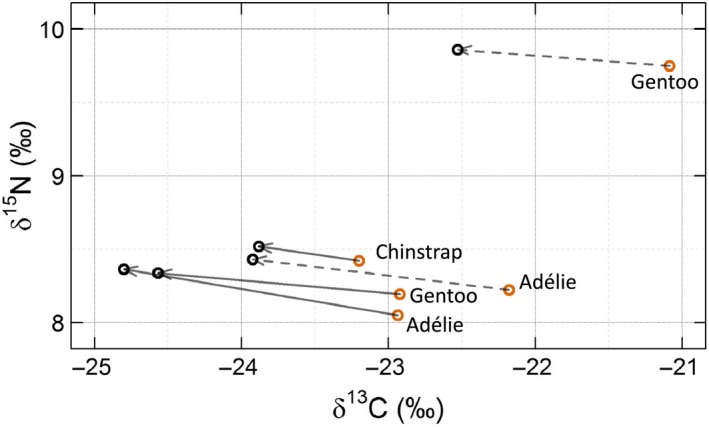
*Comparison of the shifts in mean isotopic ratios in blood (whole blood or red blood cells of adults, continuous arrows) and feather (contour feathers of chicks, dashed arrows) in pygoscelid penguins between 2014 (orange circles) and 2016 (black circles) on Powell Island. The shifts are represented as vectors in the two‐dimensional isotopic space, based on the data from Figures* 2 *and* 3

A decrease in *δ* ^13^C occurred in all three species from 2014 to 2016, both in adults and chicks (Figures 2 and 3), resulting in a shift of the Nr along the carbon axis ranging from 0.7‰ in adult chinstrap penguins to 1.9‰ in adult Adélie penguins (Figure 4). This led to a complete discrimination (i.e., 0% overlap) of the Nr from each year in the isotopic space (Figures 2and 3), except for adult chinstrap penguins where a more limited shift in *δ* ^13^C generated substantially overlapping Nr (mean overlap of Nr_2016/2014_ = 87.7%, 95% credible interval = [64.7; 99.4]). In contrast, temporal shifts in *δ* ^15^N were ≤0.3‰ for both adults and chicks (Figure 4). Overall, the amplitude of the isotopic shift was significantly different from zero in adults and chicks of all species (Figure 4; all *p* values <.001), but the amplitude of the shift was more than twice as large for adult Adélie and gentoo penguins compared to chinstrap adults (Figure 4, Table 6). The direction of the shift in the isotopic space was similar among all species and age classes (Figure 4, Table 6).

**Table 6:** Absolute differences in isotopic shift amplitude (in ‰, upper triangular matrix) and direction (in degrees, lower triangular matrix, shaded) among species and age classes in pygoscelid penguins from Powell Island, South Orkney Islands

	Adults			Chicks	
	Adélie	Chinstrap	Gentoo	Adélie	Gentoo
Adults					
Adélie	–	**1.20 (<0.001)**	0.24 (0.218)	0.13 (0.531)	**0.45 (0.032)**
Chinstrap	1.4 (0.879)	–	**0.96 (<0.001)**	**1.07 (<0.001)**	**0.75 (<0.001)**
Gentoo	4.6 (0.621)	3.2 (0.688)	–	0.10 (0.604)	0.21 (0.281)
Chicks					
Adélie	2.8 (0.776)	1.4 (0.870)	1.8 (0.845)	–	0.31 (0.135)
Gentoo	5.2 (0.605)	3.8 (0.662)	0.6 (0.952)	2.4 (0.808)	–

The isotopic shift was measured between 2014 and 2016 (Figure 3).

Empirical *p*‐values estimated from permutations procedures are in parentheses (see Section 2 for details) and in bold when significant at α = 0.05.

## 4 DISCUSSION

Our study shows that closely related species breeding in sympatry can have overlapping isotopic niches that can undergo similar variation through time, both in terms of amplitude and direction of the isotopic shift. More specifically, two main findings emerged from this study. Firstly, all three pygoscelid species had similar isotopic niche region (Nr) during the breeding season. A moderate to strong overlap was measured in the isotopic niches, and thus assumed in the trophic niches, of the three species, both in adults and chicks. This indicates that the various pygoscelid species feed, at least partly, on the same prey species in the waters around Powell Island. However, the Nr of gentoo penguins was characterized by greater variation in trophic levels with a variance in δ^15^N up to four times larger in adults. This could be a consequence of a more diverse diet among individual gentoo penguins compared to Adélie or chinstrap penguins and supporting what has been found for that species at other study sites (Camprasse, Cherel, Bustamante, Arnould, & Bost, 2017; Lescroel, Ridoux, & Bost, 2004; Polito et al., 2015; Ratcliffe & Trathan, 2011); this assertion is also borne out by diet samples collected at nearby Signy Island (BAS unpublished data; Figure 1). Secondly, a clear systemic shift in the isotopic niche of all three penguin species occurred between 2014 and 2016, in both adults and chicks. This shift was caused almost entirely by a decrease in δ^13^C, while δ^15^N values remained very similar in both years, coincidentally indicating that all three penguin species maintained a remarkably stable trophic level between these 2 years.

### 4.1 Isotopic niche width and interindividual variation in isotopic ratios

Irrespective of the species, δ^15^N measured in this study were generally moderately high, which is consistent with the contribution of prey of higher trophic level to the diet (e.g., fish or squid; Negrete et al., 2017) compared to that measured in other studies and areas. For example, stomach content analysis on chinstrap penguins from Bouvetøya described a diet composed of <1% fish during three nonconsecutive sampling years (Niemandt et al., 2016). Conversely, in the South Shetland Islands, Polito et al. (2015) found that fish contributed substantially to the diet of chinstrap and gentoo penguins alike, the latter having a diet of up to 50% fish. These authors further described that the δ^15^N values of both species were strongly and positively correlated to the estimated relative proportion of fish in the diet. It is therefore likely that the δ^15^N values seen in the current study that are in the high end of the range also correspond to higher input of fish into the penguins’ diet than those in the low end of the range. However, the absence of isotopic data on prey prevents a more precise estimation of the relative contribution of fish vs krill, for example, using Bayesian mixing models (e.g., siar; Parnell & Jackson, 2010).

Gentoo penguins showed stronger individual variation in their isotopic ratios than the two other species, with ranges in δ^15^N varying from 1.3‰ in the plasma of adults to 3.6‰ in chick feathers. A diet based on larger and older krill could induce an increase in the δ^15^N of consumers, because krill tend to increase their isotopic ratios at a rate of 0.07‰/mm as they grow (Polito, Reiss, Trivelpiece, Patterson, & Emslie, 2013). However, this would not suffice to explain all of the observed variation in the current study. It would additionally require that individuals with higher δ^15^N values had shifted their diet exclusively to larger krill. Instead, the variation in δ^15^N values documented in this study suggests a population‐level diet spanning several trophic levels, that is, a more varied diet involving stronger reliance on prey of higher trophic level, such as fish or squid. Coincidentally, this shows that, while the estimation of isotopic Nr areas constitutes a powerful and informative tool in trophic ecology, this approach might be misleading when used in isolation from other approaches to assessing trophic relationships, such as comparing intrapopulation variances in isotopic ratios (Bearhop, Adams, Waldron, Fuller, & Macleod, 2004). During the period considered in the present study (i.e., for whole blood: 2–4 weeks), individuals with high δ^15^N values were feeding more consistently at higher trophic levels, presumably on fish or squid (Miller et al., 2010). Fish and squid in the Scotia Sea and near the Antarctic Peninsula are characterized by relatively high δ^15^N values >8.0‰ (Negrete et al., 2017; Polito, Lynch et al., 2011; Polito, Trivelpiece et al., 2011). In comparison, average δ^15^N values of krill in the same region are typically <4.0‰ (review in Polito et al., 2013). Specific preservation methods used in this study can prevent a direct, quantitative comparison of the absolute isotopic ratios to those from other studies. Nevertheless, the larger variance along the nitrogen axis for gentoo penguins shows that there is high heterogeneity in the dietary habits of gentoo penguins at a population level. Gentoo penguins in other regions have also been shown to display greater foraging flexibility during the breeding season than closely related species (Lescroel et al., 2004; Miller, Karnovsky, & Trivelpiece, 2009; Polito et al., 2015; but see Juares et al., 2016; Negrete et al., 2017).

Individual variation in δ^15^N was also high in gentoo chicks, similar to their adult conspecifics. When not considering the gentoo chick that had higher δ^15^N than the others in 2016 (Figure 3), the Nr_area_ and range in δ^15^N values still remained higher for gentoo chicks as a group that year. This confirmed a generally more diversified isotopic niche for this species. In contrast, Adélie and chinstrap chicks showed much lower interindividual variation. Remarkably, the isotopic niche region of gentoo chicks also exhibited only marginal overlap with those of the two other species and indicated a diet at a higher trophic level. This contrasted with the pattern observed in gentoo adults. This could indicate stronger trophic segregation in gentoo chicks and suggests that some gentoo adults might feed their chicks with different prey than those they themselves feed on. Chick provisioning with different prey than that eaten by adults has been documented in other penguin species and can increase chicks’ growth rate when prey of higher quality are provided (Cherel, 2008). Individual specialization on particular prey types can occur in gentoo penguins (Waluda, Hill, Peat, & Trathan, 2016), which is possibly a mechanism that could help buffer intraspecific competition. The results from this study suggest that this mechanism might also apply to chick provisioning; further investigation of this hypothesis is warranted.

### 4.2 Isotopic niche overlap among species

This study adds to the growing literature supporting a potentially high level of trophic overlap in adult pygoscelid penguins (Gorman, 2015; Juares et al., 2016; Miller et al., 2010; Trivelpiece et al., 1987). This pattern was somehow moderated in chinstrap penguins, whose isotopic niches showed lower overlap with the other species’ isotopic niches in both years. The reliance of all species on the same trophic level, presumably predominantly on krill (Niemandt et al., 2016; Ratcliffe & Trathan, 2011), was clear in the present study for both years. At nearby breeding sites in the South Orkney Islands, some studies have found that krill dominates the diet of Adélie and chinstrap penguins, with estimated contributions generally over 90%, while fish seem to dominate in the diet of gentoo penguins (Lynnes, Reid, & Croxall, 2004; Rombolá, Marschoff, & Coria, 2006; White & Conroy, 1975). In other regions, the pattern seems to be similar for Adélie and chinstrap penguins (i.e., a diet almost entirely composed of krill) but much more variable for gentoo penguins, although fish seem to always contribute substantially to their diet (Bengtson, Croll, & Goebel, 1993; Lescroel et al., 2004; Miller et al., 2010; Polito et al., 2015).

The main difference between the isotopic niches of chinstrap vs Adélie and gentoo penguins was the large variation in individual δ^13^C in the former species. Variability in δ^13^C values in marine organisms can be associated with distance from shore and whether the organism feeds in the pelagic (Cherel & Hobson, 2007; Hobson et al., 1994; Kopp, Lefebvre, Cachera, Villanueva, & Ernande, 2015) or benthic food webs. Chinstrap penguins appeared to use a wider range of foraging habitats than the two other species, although this was not directly reflected in their individual δ^13^C values. Indeed, concurrent with this study on Powell Island, chinstrap penguins instrumented with GPS loggers showed a clear relationship between a strong coastal downwelling signal during the 2016 season and their movements, foraging up to 100 km farther offshore compared to birds tracked in 2014 (A. D. Lowther, P. N. Trathan, A. Tarroux, C. Lydersen, & K. M. Kovacs, in review). Krill are not passive organisms; they can move against currents, as well as migrating vertically over considerable depth ranges (Murphy et al., 1998; Tarling & Thorpe, 2014), while feeding upon diatoms which are passively transported. Consequently, the variation in δ^13^C values detected in 2016 coupled with relatively stable δ^15^N values might reflect some penguins looking for krill which in turn were searching for diatoms that were passively advected away from the shelf via coastally driven oceanographic processes. Regardless of the mechanism driving greater δ^13^C variability during 2016, the present study's results show clearly that pygoscelid penguins at Powell Island depended on similar trophic‐level prey during both years and that the isotopic shift was likely due to a shift in the carbon sources at the base of the penguins’ food chain, rather than a change in prey species.

The observed asymmetry in isotopic niche overlaps between chinstrap penguins on the one hand and Adélie and gentoo penguins on the other hand hints at behavioral mechanisms in chinstrap penguins that could potentially mitigate their competition with other pygoscelid species, when resources are limiting. Such similarities in prey used by pygoscelid penguins that forage in different habitats have been observed in other areas such as the South Shetland Islands (Kokubun, Takahashi, Mori, Watanabe, & Shin, 2010), as well as in other congeneric penguin species (Cherel, Hobson, Guinet, & Vanpe, 2007). Despite large intrapopulation variation in the δ^15^N measured in adult gentoo penguins, their isotopic niches overlapped substantially with those of both Adélie and chinstrap penguins, especially in 2014. Gentoo penguin individuals thus mostly foraged within the trophic niche of their congeneric neighbors, while only a small proportion of individuals were feeding on different prey. Overall, such findings may have important implications in term of conservation, given their potential consequences on the respective population dynamics of each species. Local gentoo penguin populations, being composed of individuals that target different prey species, might be better able to adjust their foraging tactics to potential changes in prey availability in the future compared to Adélie and chinstrap penguins, despite an apparently limited ability to forage farther from the colony in gentoo penguins (Wilson, 2010). There is also evidence that interference competition among pygoscelids occurs at least to some degree during years of low prey abundance, inducing for example spatial segregation of foraging areas and lower reproductive success (Lynnes et al., 2002). Therefore, the abundance level of prey generally available to pygoscelid species at a given breeding site but also their relative ability to adjust their diet or exclude each other from their foraging areas have direct consequences on their short‐term reproductive success and thus also on longer‐term population dynamics (Lynnes et al., 2004). At a regional scale, populations of the various pygoscelid species are experiencing differing trends (Trathan, Lynch, & Fraser, 2016); Adélie penguin populations are generally increasing in most regions of the Antarctic apart from the Peninsula where populations have declined in recent years, but now are more stable (BirdLife International 2016; Fountain et al., 2016); chinstrap penguins are stable or in decline at many locations (BirdLife International 2016); while gentoo penguins are generally increasing, particularly in the Peninsula region (BirdLife International 2016). Interspecific differences in the ability to cope with changes in environmental factors that are ultimately linked to the abundance of food resources, such as sea ice extent and duration (Rombolá, Marschoff, & Coria, 2003; Trathan et al., 1996), could at least partly explain such trends. The recent population trends at Powell Island are not currently known (Harris et al., 2015; Poncet & Poncet, 1985); however, over the past decades at the neighboring Signy Island, the number of breeding pairs of Adélie and chinstrap penguins has steadily decreased, while the number of gentoo penguins has increased (Dunn et al., 2016). This contrasts with the global population trends for chinstrap and gentoo penguins and emphasizes the need for more detailed local studies given the spatially heterogeneous response of individual populations (Hinke, Salwicka, Trivelpiece, Watters, & Trivelpiece, 2007; Lynch, Naveen, Trathan, & Fagan, 2012).

### 4.3 Interannual variation in isotopic niche

A clear alteration of the isotopic niche occurred between 2014 and 2016 in all three penguin species for both adults and chicks. Although of lesser amplitude in chinstrap penguins, this isotopic change was reflected similarly in adults and chicks of all species alike, based almost entirely on a negative shift in δ^13^C, while the δ^15^N values remained stable within all species. Several explanations are possible for these results. Firstly, all three species may have acquired resources from a different food web in 2016, for example feeding in more pelagic waters and on different species. However, the fact that δ^15^N values remained virtually unchanged makes the possibility of a clear shift in prey species unlikely (Juares et al., 2016), unless the new prey was at a very similar trophic level to those eaten in the first period. An alternate explanation is the observed shift could be the result of an environmental change between the 2 years, which might have affected the base levels of the food web, that is, the isotopic signature of either phytoplankton or particulate organic matter. However, it is not possible to confirm this latter hypothesis owing to the lack of complementary isotopic data from lower trophic levels. Independent from the origin of the change (shift in prey or change in the baseline isotopic levels), the results of this study show that all species reacted to this change similarly, although the change was weaker in chinstrap penguins. Indeed, the isotopic niche of chinstrap penguins in 2016 was nearly completely confined within that of 2014. This demonstrates that, in 2016, chinstrap penguins exclusively exploited a lesser part of the isotopic niche that they were using in 2014, simply contracting their isotopic niche.

### 4.4 Conclusion and limitations

Competition for food resources among pygoscelid penguins is expected to be particularly strong owing to their phylogenic and ecological proximity (Wilson, 2010). Using stable isotope analyses, this study showed that all three pygoscelid species had partially overlapping isotopic niches, which could be interpreted as a likely overlap in their realized trophic niches. These results support findings from previous studies showing that a high degree of reliance on the same prey species may be buffered by fine‐scale behavioral adjustments leading to the partitioning among pygoscelid penguins of their available foraging habitat (Cimino et al., 2016; Wilson, 2010). Such adjustments in foraging behavior, in combination with subtle variation in prey selection among the three species (this study; Polito et al., 2015) and distinct breeding phenologies (Ancel et al., 2013; Black, 2016), appear to be sufficient to allow the co‐occurrence of all three species breeding in sympatry and in relatively high numbers in the South Orkney Islands area. It is important to note that owing to the lack of data on resource availability it was not possible to assess the actual degree of competition among the three species in the current study. However, isotopic niche overlap, as a proxy of the trophic niche overlap, informs us about the potential for competition (Hinke et al., 2015). Importantly, our results and interpretations rely on the assumption that there are no physiological differences among individuals and species that could bias the measurements of isotopic ratios.

As stated plainly by Boersma (2008), “*Life is not likely to get easier for penguins*”: future environmental changes affecting the Southern Ocean's food web have the potential to disrupt the delicate trophic equilibrium among these species, for instance through changes in abundance of their main prey, krill (Flores et al., 2012; Lynnes et al., 2004; Melbourne‐Thomas et al., 2016). If krill abundance was to decline drastically in the near future, the ecological similarity among pygoscelids could lead to high levels of competition for food resources (Miller et al., 2010), with uncertain outcomes. There are data suggesting that, in such a scenario, owing to their greater ecological flexibility, gentoo penguin populations may cope better than their congeners in the Antarctic Peninsula area (Carlini et al., 2009; Levy et al., 2016; Lima & Estay, 2013; Lynch, Naveen et al., 2012; Trivelpiece et al., 1987). Understanding how congeneric species breeding in sympatry can adapt to such changes is achievable through individual‐based studies of their respective isotopic niches that also integrate fluctuations of their isotopic environment and the dynamics of their foraging patterns at fine spatiotemporal scales and ultimately determining the consequences on their reproductive success and survival. The interpretation of the results from the current study is limited by the absence of isotopic data on prey. It relies solely on the interpretation of differences in isotopic ratios among consumers. Furthermore, the resolution provided by a two‐dimensional isotopic space might not be sufficient to detect changes or differences in small amplitude in the isotopic niches. Using a third isotope such as sulfur (^34^S/^32^S) might provide valuable complementary information (Bradshaw et al., 2017; Rubenstein & Hobson, 2004). Finally, complementary techniques of diet reconstruction, such as stomach content analysis (Polito et al., 2011c), should ideally be performed to confirm the trends suggested herein and to allow for the interpretation of any potential subtle changes in diet.

## CONFLICT OF INTEREST

None declared.

## AUTHOR CONTRIBUTIONS

All authors participated in the study design. KK and PT contributed to the field planning and logistics and to the laboratory analyses. PT was part of the field team on Powell in 2014. AT prepared the tissue samples in the laboratory, formatted the raw isotopic data, ran the statistical analyses, produced the figures and wrote the manuscript. All authors commented upon the manuscript in multiple draft rounds, thus contributing critically to the development and production of the manuscript.
