# Supplementary material for: Temporal variation in trophic relationships among three congeneric penguin species breeding in sympatry
Source: Ecol Evol. 2018 Mar 5;8(7):3660–74. doi: 10.1002/ece3.3937 (PMC5901183; doi:10.1002/ece3.3937)
Supplement: Supplementary file 4 [file ECE3-8-3660-s004.docx]

**SUPPLEMENTARY FIGURE LEGENDS**

**Figure S1 –** Temporal variation in δ^13^C in blood (left panel) and plasma (right panel) in adult Adélie, Chinstrap, and Gentoo penguins from Powell Island, South Orkney Islands in 2014 (orange) and 2016 (black). The temporal scale on the X-axis is continuous, with day as unit. Plasma was not sampled in 2014. Intra-seasonal temporal trends were tested using simple linear models whenever possible (continuous lines ±95% CI as shaded areas), i.e. when the temporal coverage throughout the season was appropriate (only for Chinstrap and Gentoo penguins in 2016).

**Figure S2 –** Temporal variation in δ^15^N in blood (left panel) and plasma (right panel) in adult Adélie, Chinstrap, and Gentoo penguins from Powell Island, South Orkney Islands in 2014 (orange) and 2016 (black). The temporal scale on the X-axis is continuous, with day as unit. Plasma was not sampled in 2014. Intra-seasonal temporal trends were tested using simple linear models whenever possible (continuous lines ±95% CI as shaded areas), i.e. when the temporal coverage throughout the season was appropriate (only for Chinstrap and Gentoo penguins in 2016).

**Figure S3 –** Temporal variation in δ^13^C of feathers (contour feathers) and down (down feathers) of chick Adélie, Chinstrap, and Gentoo penguins from Powell Island, South Orkney Islands in 2014 (orange) and 2016 (black). The temporal scale on the X-axis is continuous, with day as unit. Chinstrap chicks were not sampled in 2016.

**SUPPLEMENTARY TABLES**

|  |  | Adults |  |  | Chicks |  |
| --- | --- | --- | --- | --- | --- | --- |
|  |  | Blood | Plasma |  | Down | Feather |
| 2014 | Adélie | 15 | - |  | 23 | 22 |
|  | Chinstrap | 16 | - |  | 25 | 25 |
|  | Gentoo | 15 | - |  | 24 | 24 |
| 2016 | Adélie | 12 | 10 |  | 10 | 10 |
|  | Chinstrap | 46 | 33 |  | - | - |
|  | Gentoo | 22 | 15 |  | 10 | 9 |

**Table S1 –** Sampling scheme of isotopic data: sample size per breeding season, species, age class, and tissue. Dashes indicate when no samples were available. ‘Blood’ stands for ‘Whole blood’ or ‘Red blood cells’ (see methods for details). ‘Feather’ stands for ‘Contour feather’. Note that some plasma samples from 2016 were too small to be processed and analysed for isotopic ratios. Similarly, two feather samples from chicks could not be analysed.

| **Bulk** | | |  | **Lipid extracted** | | |  | **Difference** | | |
| --- | --- | --- | --- | --- | --- | --- | --- | --- | --- | --- |
| **δ^13^C** | **δ^15^N** | **C:N ratio** |  | **δ^13^C** | **δ^15^N** | **C:N ratio** |  | **Δδ^13^C** | **Δδ^15^N** | **ΔC:N ratio** |
| -25.8 | 8.5 | 3.9 |  | -24.7 | 8.6 | 3.3 |  | 1.1 | 0.1 | -0.6 |
| -25.2 | 8.4 | 3.7 |  | -24.3 | 8.5 | 3.2 |  | 0.9 | 0.1 | -0.5 |
| -25.2 | 8.7 | 3.9 |  | -24.3 | 8.9 | 3.3 |  | 0.9 | 0.2 | -0.6 |
| -25.6 | 9.8 | 3.8 |  | -24.5 | 9.8 | 3.3 |  | 1.1 | 0 | -0.5 |
| -26.0 | 8.8 | 4.3 |  | -24.6 | 9.1 | 3.4 |  | 1.4 | 0.3 | -0.9 |
| -25.8 | 9.0 | 3.8 |  | -24.9 | 8.8 | 3.4 |  | 0.9 | -0.2 | -0.5 |
| -25.2 | 8.5 | 3.7 |  | -24.5 | 8.5 | 3.3 |  | 0.8 | 0 | -0.5 |
| -26.1 | 8.9 | 4.0 |  | -24.9 | 9.0 | 3.4 |  | 1.2 | 0.1 | -0.6 |
| -25.0 | 8.7 | 3.7 |  | -24.1 | 8.6 | 3.3 |  | 0.9 | -0.1 | -0.5 |
| -25.7 | 8.9 | 4.2 |  | -24.4 | 9.0 | 3.4 |  | 1.3 | 0.2 | -0.8 |
| -24.4 | 8.7 | 3.8 |  | -23.5 | 8.8 | 3.4 |  | 0.9 | 0.1 | -0.5 |
| -25.0 | 8.7 | 4.0 |  | -23.9 | 8.9 | 3.4 |  | 1.1 | 0.2 | -0.6 |
| -24.4 | 9.1 | 3.8 |  | -23.2 | 9.3 | 3.3 |  | 1.2 | 0.2 | -0.5 |
| -24.8 | 8.9 | 4.2 |  | -23.4 | 9.2 | 3.4 |  | 1.4 | 0.3 | -0.7 |
| -24.0 | 9.5 | 3.6 |  | -23.3 | 9.4 | 3.3 |  | 0.8 | 0 | -0.3 |
| -24.5 | 8.8 | 3.6 |  | -24.3 | 8.9 | 3.3 |  | 0.3 | 0.1 | -0.3 |
| -25.0 | 9.1 | 4.0 |  | -23.7 | 9.2 | 3.3 |  | 1.3 | 0 | -0.7 |
| -28.0 | 8.4 | 6.7 |  | -25.1 | 9.1 | 3.8 |  | 2.9 | 0.6 | -2.9 |
| -24.5 | 8.9 | 3.6 |  | -23.7 | 8.7 | 3.3 |  | 0.8 | -0.2 | -0.4 |
| -23.7 | 8.7 | 3.5 |  | -23.1 | 8.5 | 3.3 |  | 0.6 | -0.1 | -0.2 |
| -24.3 | 9.0 | 3.8 |  | -23.5 | 9.0 | 3.3 |  | 0.8 | 0 | -0.5 |
| -25.2 | 8.4 | 3.9 |  | -24.1 | 8.5 | 3.3 |  | 1.1 | 0.1 | -0.6 |
| -25.1 | 8.5 | 4.0 |  | -23.9 | 8.6 | 3.4 |  | 1.2 | 0.1 | -0.6 |
| -24.7 | 8.4 | 3.7 |  | -24.0 | 8.4 | 3.3 |  | 0.7 | 0 | -0.4 |
| -23.6 | 8.5 | 3.2 |  | -23.3 | 8.4 | 3.2 |  | 0.4 | -0.2 | 0 |
| -24.7 | 8.1 | 3.8 |  | -23.4 | 8.2 | 3.2 |  | 1.3 | 0.1 | -0.6 |
| -23.7 | 8.3 | 3.4 |  | -23.3 | 8.2 | 3.2 |  | 0.4 | 0 | -0.2 |
| -24.5 | 8.1 | 3.7 |  | -23.8 | 8.0 | 3.3 |  | 0.7 | 0 | -0.4 |
| -24.5 | 8.5 | 3.7 |  | -23.7 | 8.5 | 3.3 |  | 0.8 | 0 | -0.4 |
| -25.3 | 8.3 | 3.7 |  | -24.3 | 8.4 | 3.3 |  | 1.0 | 0.1 | -0.4 |
| -25.0 | 8.3 | 3.5 |  | -24.4 | 8.3 | 3.2 |  | 0.6 | 0.1 | -0.3 |
| -24.7 | 8.3 | 3.3 |  | -24.4 | 8.2 | 3.2 |  | 0.3 | -0.1 | -0.1 |
| -24.5 | 8.9 | 3.3 |  | -24.4 | 8.8 | 3.2 |  | 0.1 | -0.1 | 0 |
| -25.1 | 9.4 | 3.8 |  | -24.2 | 9.4 | 3.3 |  | 0.8 | 0.1 | -0.5 |
| -25.3 | 8.4 | 3.7 |  | -24.4 | 8.5 | 3.3 |  | 0.9 | 0.1 | -0.4 |
| -25.0 | 8.3 | 3.5 |  | -24.5 | 8.3 | 3.2 |  | 0.6 | -0.1 | -0.2 |
| -25.5 | 9.1 | 3.9 |  | -24.5 | 9.2 | 3.3 |  | 1.0 | 0.0 | -0.6 |
| -25.3 | 8.5 | 3.6 |  | -24.5 | 8.6 | 3.3 |  | 0.8 | 0.1 | -0.3 |
| -24.4 | 8.7 | 3.6 |  | -23.8 | 8.8 | 3.3 |  | 0.7 | 0.1 | -0.3 |
| -24.6 | 8.5 | 3.7 |  | -23.9 | 8.5 | 3.3 |  | 0.7 | 0.0 | -0.4 |
| -24.5 | 8.1 | 3.4 |  | -24.1 | 8.2 | 3.2 |  | 0.5 | 0.0 | -0.2 |
| -24.5 | 8.2 | 3.8 |  | -23.7 | 8.3 | 3.4 |  | 0.8 | 0.1 | -0.4 |
| -25.8 | 9.4 | 5.4 |  | -23.9 | 9.8 | 3.5 |  | 1.9 | 0.4 | -1.9 |
| -25.6 | 9.1 | 5.0 |  | -24.2 | 9.2 | 3.6 |  | 1.4 | 0.1 | -1.4 |
| -26.4 | 8.7 | 6.0 |  | -24.4 | 9.1 | 3.7 |  | 2.0 | 0.4 | -2.2 |
| -27.1 | 8.4 | 8.0 |  | -24.6 | 8.9 | 4.0 |  | 2.5 | 0.4 | -4.0 |

**Table S2.** Carbon and nitrogen isotopic ratios of bulk vs delipidated plasma samples (N = 46) from pygoscelid penguins from Powell Island, that were used to derive normalization equations specific to our study system.

| **Species** | **Age** | **Tissue** | **Collection**  **date** | ***δ*^13^C**  **bulk** | ***δ*^15^N**  **bulk** | **C:N ratio bulk** | ***δ*^13^C corrected** |
| --- | --- | --- | --- | --- | --- | --- | --- |
| Adélie | Adult | Blood whole | 2014-02-03 | -22.7 | 8.0 | 3.2 | n/a |
| Adélie | Adult | Blood whole | 2014-02-03 | -22.7 | 8.1 | 3.2 | n/a |
| Adélie | Adult | Blood whole | 2014-02-03 | -23.0 | 8.0 | 3.2 | n/a |
| Adélie | Adult | Blood whole | 2014-02-03 | -22.6 | 7.7 | 3.2 | n/a |
| Adélie | Adult | Blood whole | 2014-02-03 | -23.3 | 8.5 | 3.2 | n/a |
| Adélie | Adult | Blood whole | 2014-02-03 | -22.7 | 8.8 | 3.2 | n/a |
| Adélie | Adult | Blood whole | 2014-02-03 | -23.0 | 7.7 | 3.3 | n/a |
| Adélie | Adult | Blood whole | 2014-02-03 | -23.0 | 8.0 | 3.2 | n/a |
| Adélie | Adult | Blood whole | 2014-02-03 | -23.1 | 8.3 | 3.2 | n/a |
| Adélie | Adult | Blood whole | 2014-02-03 | -22.7 | 8.1 | 3.2 | n/a |
| Adélie | Adult | Blood whole | 2014-02-03 | -22.8 | 8.0 | 3.2 | n/a |
| Adélie | Adult | Blood whole | 2014-02-03 | -23.0 | 7.9 | 3.2 | n/a |
| Adélie | Adult | Blood whole | 2014-02-03 | -23.3 | 7.9 | 3.2 | n/a |
| Adélie | Adult | Blood whole | 2014-02-03 | -22.8 | 7.8 | 3.2 | n/a |
| Adélie | Adult | Blood whole | 2014-02-03 | -23.3 | 7.9 | 3.2 | n/a |
| Adélie | Adult | Plasma | 2015-12-29 | -25.8 | 8.5 | 3.9 | -24.7 |
| Adélie | Adult | Plasma | 2015-12-30 | -25.2 | 8.4 | 3.7 | -24.3 |
| Adélie | Adult | Plasma | 2015-12-31 | -25.2 | 8.7 | 3.9 | -24.2 |
| Adélie | Adult | Plasma | 2015-12-31 | -25.6 | 9.8 | 3.8 | -24.7 |
| Adélie | Adult | Plasma | 2016-01-01 | -26.0 | 8.8 | 4.3 | -24.6 |
| Adélie | Adult | Plasma | 2016-01-01 | -25.8 | 9.0 | 3.8 | -24.9 |
| Adélie | Adult | Plasma | 2016-01-02 | -25.2 | 8.5 | 3.7 | -24.4 |
| Adélie | Adult | Plasma | 2016-01-02 | -26.1 | 8.9 | 4.0 | -25.0 |
| Adélie | Adult | Plasma | 2016-01-02 | -25.0 | 8.7 | 3.7 | -24.1 |
| Adélie | Adult | Plasma | 2016-01-03 | -25.7 | 8.9 | 4.2 | -24.4 |
| Adélie | Adult | Red blood cells | 2015-12-29 | -25.2 | 8.3 | 3.3 | n/a |
| Adélie | Adult | Red blood cells | 2015-12-30 | -24.6 | 8.1 | 3.2 | n/a |
| Adélie | Adult | Red blood cells | 2015-12-31 | -24.7 | 8.4 | 3.3 | n/a |
| Adélie | Adult | Red blood cells | 2015-12-31 | -25.0 | 8.7 | 3.3 | n/a |
| Adélie | Adult | Red blood cells | 2016-01-01 | -24.4 | 8.3 | 3.2 | n/a |
| Adélie | Adult | Red blood cells | 2016-01-01 | -25.3 | 8.5 | 3.3 | n/a |
| Adélie | Adult | Red blood cells | 2016-01-01 | -24.8 | 8.5 | 3.3 | n/a |
| Adélie | Adult | Red blood cells | 2016-01-02 | -24.8 | 8.1 | 3.2 | n/a |
| Adélie | Adult | Red blood cells | 2016-01-02 | -24.9 | 8.6 | 3.2 | n/a |
| Adélie | Adult | Red blood cells | 2016-01-02 | -24.4 | 8.0 | 3.2 | n/a |
| Adélie | Adult | Red blood cells | 2016-01-02 | -24.7 | 8.0 | 3.2 | n/a |
| Adélie | Adult | Red blood cells | 2016-01-03 | -24.7 | 8.8 | 3.2 | n/a |
| Adélie | Chick | Down | 2014-02-10 | -22.5 | 8.4 | 3.0 | n/a |
| Adélie | Chick | Down | 2014-02-10 | -22.5 | 8.1 | 3.1 | n/a |
| Adélie | Chick | Down | 2014-02-10 | -22.6 | 7.8 | 3.0 | n/a |
| Adélie | Chick | Down | 2014-02-10 | -22.4 | 7.9 | 3.1 | n/a |
| Adélie | Chick | Down | 2014-02-10 | -22.6 | 7.9 | 3.0 | n/a |
| Adélie | Chick | Down | 2014-02-10 | -22.7 | 7.3 | 2.8 | n/a |
| Adélie | Chick | Down | 2014-02-10 | -22.0 | 8.7 | 3.0 | n/a |
| Adélie | Chick | Down | 2014-02-10 | -22.3 | 7.4 | 3.1 | n/a |
| Adélie | Chick | Down | 2014-02-10 | -22.3 | 7.6 | 3.1 | n/a |
| Adélie | Chick | Down | 2014-02-10 | -22.5 | 7.8 | 3.1 | n/a |
| Adélie | Chick | Down | 2014-02-10 | -22.4 | 7.6 | 3.1 | n/a |
| Adélie | Chick | Down | 2014-02-10 | -22.3 | 7.9 | 3.1 | n/a |
| Adélie | Chick | Down | 2014-02-10 | -22.5 | 8.2 | 3.1 | n/a |
| Adélie | Chick | Down | 2014-02-10 | -22.3 | 8.3 | 3.1 | n/a |
| Adélie | Chick | Down | 2014-02-10 | -22.1 | 7.9 | 3.0 | n/a |
| Adélie | Chick | Down | 2014-02-10 | -22.8 | 7.9 | 3.1 | n/a |
| Adélie | Chick | Down | 2014-02-10 | -22.5 | 8.1 | 3.1 | n/a |
| Adélie | Chick | Down | 2014-02-10 | -22.2 | 8.1 | 3.1 | n/a |
| Adélie | Chick | Down | 2014-02-10 | -22.1 | 8.1 | 3.0 | n/a |
| Adélie | Chick | Down | 2014-02-10 | -22.3 | 8.3 | 3.1 | n/a |
| Adélie | Chick | Down | 2014-02-10 | -22.3 | 7.9 | 3.0 | n/a |
| Adélie | Chick | Down | 2014-02-10 | -22.3 | 7.7 | 2.9 | n/a |
| Adélie | Chick | Down | 2014-02-10 | -22.6 | 8.0 | 2.5 | n/a |
| Adélie | Chick | Down | 2016-02-03 | -24.3 | 8.2 | 3.1 | n/a |
| Adélie | Chick | Down | 2016-02-03 | -24.2 | 8.5 | 3.1 | n/a |
| Adélie | Chick | Down | 2016-02-03 | -24.4 | 8.7 | 3.1 | n/a |
| Adélie | Chick | Down | 2016-02-03 | -24.5 | 8.7 | 3.1 | n/a |
| Adélie | Chick | Down | 2016-02-03 | -24.5 | 8.7 | 3.1 | n/a |
| Adélie | Chick | Down | 2016-02-03 | -24.6 | 8.6 | 2.9 | n/a |
| Adélie | Chick | Down | 2016-02-03 | -24.1 | 7.7 | 2.7 | n/a |
| Adélie | Chick | Down | 2016-02-03 | -24.3 | 8.4 | 3.2 | n/a |
| Adélie | Chick | Down | 2016-02-03 | -24.6 | 8.2 | 3.0 | n/a |
| Adélie | Chick | Down | 2016-02-03 | -24.2 | 7.8 | 2.8 | n/a |
| Adélie | Chick | Feather | 2014-02-10 | -22.1 | 8.4 | 3.1 | n/a |
| Adélie | Chick | Feather | 2014-02-10 | -22.1 | 8.4 | 3.1 | n/a |
| Adélie | Chick | Feather | 2014-02-10 | -21.9 | 7.9 | 3.1 | n/a |
| Adélie | Chick | Feather | 2014-02-10 | -22.4 | 8.5 | 3.1 | n/a |
| Adélie | Chick | Feather | 2014-02-10 | -22.1 | 8.1 | 3.1 | n/a |
| Adélie | Chick | Feather | 2014-02-10 | -22.1 | 7.9 | 3.1 | n/a |
| Adélie | Chick | Feather | 2014-02-10 | -22.2 | 8.7 | 3.1 | n/a |
| Adélie | Chick | Feather | 2014-02-10 | -21.8 | 8.0 | 3.1 | n/a |
| Adélie | Chick | Feather | 2014-02-10 | -22.1 | 8.0 | 3.1 | n/a |
| Adélie | Chick | Feather | 2014-02-10 | -22.3 | 8.4 | 3.1 | n/a |
| Adélie | Chick | Feather | 2014-02-10 | -22.1 | 8.0 | 3.1 | n/a |
| Adélie | Chick | Feather | 2014-02-10 | -22.4 | 8.3 | 3.1 | n/a |
| Adélie | Chick | Feather | 2014-02-10 | -22.2 | 8.4 | 3.1 | n/a |
| Adélie | Chick | Feather | 2014-02-10 | -22.1 | 8.5 | 3.1 | n/a |
| Adélie | Chick | Feather | 2014-02-10 | -22.2 | 8.1 | 3.1 | n/a |
| Adélie | Chick | Feather | 2014-02-10 | -22.6 | 8.5 | 3.1 | n/a |
| Adélie | Chick | Feather | 2014-02-10 | -22.1 | 8.4 | 3.1 | n/a |
| Adélie | Chick | Feather | 2014-02-10 | -22.6 | 8.4 | 3.1 | n/a |
| Adélie | Chick | Feather | 2014-02-10 | -22.1 | 7.9 | 3.1 | n/a |
| Adélie | Chick | Feather | 2014-02-10 | -22.1 | 8.4 | 3.1 | n/a |
| Adélie | Chick | Feather | 2014-02-10 | -22.1 | 8.1 | 3.1 | n/a |
| Adélie | Chick | Feather | 2014-02-10 | -22.2 | 7.7 | 3.1 | n/a |
| Adélie | Chick | Feather | 2016-02-03 | -23.9 | 8.5 | 3.1 | n/a |
| Adélie | Chick | Feather | 2016-02-03 | -23.8 | 8.6 | 3.1 | n/a |
| Adélie | Chick | Feather | 2016-02-03 | -23.7 | 8.3 | 3.1 | n/a |
| Adélie | Chick | Feather | 2016-02-03 | -23.3 | 8.7 | 3.2 | n/a |
| Adélie | Chick | Feather | 2016-02-03 | -24.0 | 8.5 | 3.2 | n/a |
| Adélie | Chick | Feather | 2016-02-03 | -24.3 | 8.6 | 3.1 | n/a |
| Adélie | Chick | Feather | 2016-02-03 | -24.1 | 8.4 | 3.1 | n/a |
| Adélie | Chick | Feather | 2016-02-03 | -24.0 | 8.3 | 3.2 | n/a |
| Adélie | Chick | Feather | 2016-02-03 | -23.9 | 8.1 | 3.1 | n/a |
| Adélie | Chick | Feather | 2016-02-03 | -24.2 | 8.3 | 3.1 | n/a |
| Chinstrap | Adult | Blood whole | 2014-02-02 | -22.6 | 8.4 | 3.2 | n/a |
| Chinstrap | Adult | Blood whole | 2014-02-02 | -22.7 | 8.6 | 3.1 | n/a |
| Chinstrap | Adult | Blood whole | 2014-02-02 | -23.0 | 8.3 | 3.2 | n/a |
| Chinstrap | Adult | Blood whole | 2014-02-02 | -23.3 | 8.3 | 3.1 | n/a |
| Chinstrap | Adult | Blood whole | 2014-02-02 | -23.5 | 8.9 | 3.1 | n/a |
| Chinstrap | Adult | Blood whole | 2014-02-02 | -22.6 | 8.5 | 3.1 | n/a |
| Chinstrap | Adult | Blood whole | 2014-02-02 | -23.0 | 8.1 | 3.1 | n/a |
| Chinstrap | Adult | Blood whole | 2014-02-02 | -23.8 | 8.2 | 3.1 | n/a |
| Chinstrap | Adult | Blood whole | 2014-02-02 | -22.9 | 8.5 | 3.1 | n/a |
| Chinstrap | Adult | Blood whole | 2014-02-02 | -23.0 | 8.7 | 3.1 | n/a |
| Chinstrap | Adult | Blood whole | 2014-02-02 | -22.8 | 8.5 | 3.1 | n/a |
| Chinstrap | Adult | Blood whole | 2014-02-02 | -22.8 | 8.5 | 3.1 | n/a |
| Chinstrap | Adult | Blood whole | 2014-02-02 | -22.9 | 8.2 | 3.1 | n/a |
| Chinstrap | Adult | Blood whole | 2014-02-02 | -23.8 | 8.2 | 3.2 | n/a |
| Chinstrap | Adult | Blood whole | 2014-02-02 | -23.7 | 8.7 | 3.1 | n/a |
| Chinstrap | Adult | Blood whole | 2014-02-02 | -24.7 | 8.2 | 3.1 | n/a |
| Chinstrap | Adult | Plasma | 2015-12-24 | -24.4 | 8.7 | 3.8 | -23.4 |
| Chinstrap | Adult | Plasma | 2015-12-24 | -24.4 | 9.0 | 4.0 | -23.3 |
| Chinstrap | Adult | Plasma | 2015-12-24 | -24.4 | 9.1 | 3.8 | -23.5 |
| Chinstrap | Adult | Plasma | 2015-12-24 | -25.4 | 9.0 | 3.9 | -24.4 |
| Chinstrap | Adult | Plasma | 2015-12-28 | -25.0 | 8.7 | 4.0 | -23.9 |
| Chinstrap | Adult | Plasma | 2015-12-28 | -25.4 | 8.5 | 4.4 | -23.9 |
| Chinstrap | Adult | Plasma | 2015-12-28 | -24.8 | 8.9 | 4.2 | -23.5 |
| Chinstrap | Adult | Plasma | 2015-12-29 | -24.3 | 9.1 | 3.9 | -23.2 |
| Chinstrap | Adult | Plasma | 2015-12-29 | -24.1 | 9.1 | 3.9 | -23.0 |
| Chinstrap | Adult | Plasma | 2016-01-02 | -25.5 | 8.6 | 3.8 | -24.6 |
| Chinstrap | Adult | Plasma | 2016-01-02 | -25.5 | 8.7 | 4.2 | -24.2 |
| Chinstrap | Adult | Plasma | 2016-01-05 | -25.3 | 8.3 | 3.7 | -24.4 |
| Chinstrap | Adult | Plasma | 2016-01-05 | -24.6 | 8.7 | 3.9 | -23.5 |
| Chinstrap | Adult | Plasma | 2016-01-07 | -24.7 | 8.5 | 4.2 | -23.4 |
| Chinstrap | Adult | Plasma | 2016-01-11 | -25.4 | 8.8 | 4.0 | -24.2 |
| Chinstrap | Adult | Plasma | 2016-01-16 | -24.7 | 8.5 | 3.7 | -23.9 |
| Chinstrap | Adult | Plasma | 2016-01-16 | -24.0 | 9.5 | 3.6 | -23.3 |
| Chinstrap | Adult | Plasma | 2016-01-16 | -24.3 | 8.6 | 3.8 | -23.4 |
| Chinstrap | Adult | Plasma | 2016-01-16 | -24.5 | 8.8 | 3.6 | -23.9 |
| Chinstrap | Adult | Plasma | 2016-01-18 | -25.0 | 9.1 | 4.0 | -23.8 |
| Chinstrap | Adult | Plasma | 2016-01-19 | -24.5 | 8.7 | 3.6 | -23.7 |
| Chinstrap | Adult | Plasma | 2016-01-22 | -28.0 | 8.4 | 6.7 | -25.6 |
| Chinstrap | Adult | Plasma | 2016-01-22 | -24.5 | 8.9 | 3.6 | -23.7 |
| Chinstrap | Adult | Plasma | 2016-01-22 | -24.1 | 8.5 | 3.5 | -23.5 |
| Chinstrap | Adult | Plasma | 2016-01-22 | -25.1 | 8.5 | 4.0 | -24.0 |
| Chinstrap | Adult | Plasma | 2016-01-23 | -23.7 | 8.7 | 3.5 | -23.1 |
| Chinstrap | Adult | Plasma | 2016-01-28 | -24.3 | 9.0 | 3.8 | -23.4 |
| Chinstrap | Adult | Plasma | 2016-01-28 | -25.2 | 8.4 | 3.9 | -24.2 |
| Chinstrap | Adult | Plasma | 2016-01-28 | -23.9 | 8.5 | 3.5 | -23.4 |
| Chinstrap | Adult | Plasma | 2016-01-29 | -24.5 | 8.9 | 3.8 | -23.6 |
| Chinstrap | Adult | Plasma | 2016-01-30 | -24.7 | 8.4 | 3.6 | -23.9 |
| Chinstrap | Adult | Plasma | 2016-01-30 | -24.5 | 8.5 | 3.7 | -23.7 |
| Chinstrap | Adult | Plasma | 2016-02-09 | -24.5 | 8.5 | 3.7 | -23.7 |
| Chinstrap | Adult | Red blood cells | 2015-12-22 | -23.8 | 8.9 | 3.3 | n/a |
| Chinstrap | Adult | Red blood cells | 2015-12-22 | -23.6 | 9.0 | 3.3 | n/a |
| Chinstrap | Adult | Red blood cells | 2015-12-22 | -23.6 | 8.3 | 3.4 | n/a |
| Chinstrap | Adult | Red blood cells | 2015-12-24 | -23.8 | 8.4 | 3.2 | n/a |
| Chinstrap | Adult | Red blood cells | 2015-12-24 | -24.1 | 8.8 | 3.2 | n/a |
| Chinstrap | Adult | Red blood cells | 2015-12-24 | -23.7 | 8.9 | 3.3 | n/a |
| Chinstrap | Adult | Red blood cells | 2015-12-24 | -24.1 | 9.0 | 3.2 | n/a |
| Chinstrap | Adult | Red blood cells | 2015-12-28 | -24.4 | 8.4 | 3.5 | n/a |
| Chinstrap | Adult | Red blood cells | 2015-12-28 | -23.7 | 8.6 | 3.3 | n/a |
| Chinstrap | Adult | Red blood cells | 2015-12-28 | -24.0 | 8.8 | 3.3 | n/a |
| Chinstrap | Adult | Red blood cells | 2015-12-29 | -23.9 | 9.1 | 3.2 | n/a |
| Chinstrap | Adult | Red blood cells | 2015-12-29 | -23.9 | 9.1 | 3.5 | n/a |
| Chinstrap | Adult | Red blood cells | 2015-12-29 | -24.1 | 8.7 | 3.3 | n/a |
| Chinstrap | Adult | Red blood cells | 2016-01-02 | -24.5 | 8.5 | 3.2 | n/a |
| Chinstrap | Adult | Red blood cells | 2016-01-03 | -23.8 | 8.9 | 3.2 | n/a |
| Chinstrap | Adult | Red blood cells | 2016-01-03 | -24.6 | 8.6 | 3.4 | n/a |
| Chinstrap | Adult | Red blood cells | 2016-01-05 | -24.3 | 8.3 | 3.2 | n/a |
| Chinstrap | Adult | Red blood cells | 2016-01-07 | -23.2 | 8.6 | 3.2 | n/a |
| Chinstrap | Adult | Red blood cells | 2016-01-07 | -24.0 | 8.5 | 3.2 | n/a |
| Chinstrap | Adult | Red blood cells | 2016-01-10 | -24.1 | 8.9 | 3.3 | n/a |
| Chinstrap | Adult | Red blood cells | 2016-01-11 | -24.4 | 8.6 | 3.3 | n/a |
| Chinstrap | Adult | Red blood cells | 2016-01-11 | -23.9 | 8.6 | 3.4 | n/a |
| Chinstrap | Adult | Red blood cells | 2016-01-16 | -23.7 | 8.3 | 3.2 | n/a |
| Chinstrap | Adult | Red blood cells | 2016-01-16 | -23.4 | 8.7 | 3.2 | n/a |
| Chinstrap | Adult | Red blood cells | 2016-01-16 | -23.3 | 8.3 | 3.2 | n/a |
| Chinstrap | Adult | Red blood cells | 2016-01-16 | -24.6 | 8.4 | 3.5 | n/a |
| Chinstrap | Adult | Red blood cells | 2016-01-18 | -24.0 | 8.6 | 3.4 | n/a |
| Chinstrap | Adult | Red blood cells | 2016-01-19 | -23.9 | 8.6 | 3.2 | n/a |
| Chinstrap | Adult | Red blood cells | 2016-01-20 | -23.8 | 8.1 | 3.3 | n/a |
| Chinstrap | Adult | Red blood cells | 2016-01-21 | -23.6 | 8.6 | 3.2 | n/a |
| Chinstrap | Adult | Red blood cells | 2016-01-22 | -24.5 | 8.8 | 3.3 | n/a |
| Chinstrap | Adult | Red blood cells | 2016-01-22 | -24.1 | 8.5 | 3.3 | n/a |
| Chinstrap | Adult | Red blood cells | 2016-01-22 | -23.3 | 8.3 | 3.2 | n/a |
| Chinstrap | Adult | Red blood cells | 2016-01-22 | -23.9 | 8.4 | 3.2 | n/a |
| Chinstrap | Adult | Red blood cells | 2016-01-23 | -23.1 | 8.3 | 3.2 | n/a |
| Chinstrap | Adult | Red blood cells | 2016-01-28 | -23.6 | 8.6 | 3.2 | n/a |
| Chinstrap | Adult | Red blood cells | 2016-01-28 | -23.9 | 8.0 | 3.2 | n/a |
| Chinstrap | Adult | Red blood cells | 2016-01-28 | -23.8 | 8.3 | 3.2 | n/a |
| Chinstrap | Adult | Red blood cells | 2016-01-29 | -23.7 | 8.4 | 3.2 | n/a |
| Chinstrap | Adult | Red blood cells | 2016-01-30 | -24.0 | 8.2 | 3.3 | n/a |
| Chinstrap | Adult | Red blood cells | 2016-01-30 | -23.7 | 8.2 | 3.2 | n/a |
| Chinstrap | Adult | Red blood cells | 2016-02-04 | -23.8 | 8.1 | 3.2 | n/a |
| Chinstrap | Adult | Red blood cells | 2016-02-04 | -23.9 | 8.1 | 3.3 | n/a |
| Chinstrap | Adult | Red blood cells | 2016-02-06 | -23.8 | 8.1 | 3.2 | n/a |
| Chinstrap | Adult | Red blood cells | 2016-02-09 | -23.7 | 8.3 | 3.3 | n/a |
| Chinstrap | Adult | Red blood cells | 2016-02-09 | -24.0 | 8.3 | 3.2 | n/a |
| Chinstrap | Chick | Down | 2014-02-10 | -21.8 | 8.2 | 3.1 | n/a |
| Chinstrap | Chick | Down | 2014-02-10 | -21.9 | 7.8 | 3.1 | n/a |
| Chinstrap | Chick | Down | 2014-02-10 | -22.1 | 8.2 | 3.1 | n/a |
| Chinstrap | Chick | Down | 2014-02-10 | -22.0 | 8.2 | 3.1 | n/a |
| Chinstrap | Chick | Down | 2014-02-10 | -21.8 | 7.8 | 3.1 | n/a |
| Chinstrap | Chick | Down | 2014-02-10 | -22.1 | 8.0 | 3.1 | n/a |
| Chinstrap | Chick | Down | 2014-02-10 | -22.2 | 8.1 | 3.1 | n/a |
| Chinstrap | Chick | Down | 2014-02-10 | -22.0 | 8.4 | 3.1 | n/a |
| Chinstrap | Chick | Down | 2014-02-10 | -22.1 | 8.0 | 3.1 | n/a |
| Chinstrap | Chick | Down | 2014-02-10 | -22.0 | 8.1 | 3.1 | n/a |
| Chinstrap | Chick | Down | 2014-02-10 | -21.9 | 8.1 | 3.1 | n/a |
| Chinstrap | Chick | Down | 2014-02-10 | -21.9 | 8.5 | 3.1 | n/a |
| Chinstrap | Chick | Down | 2014-02-10 | -22.0 | 8.2 | 3.1 | n/a |
| Chinstrap | Chick | Down | 2014-02-10 | -22.0 | 8.0 | 3.1 | n/a |
| Chinstrap | Chick | Down | 2014-02-10 | -22.0 | 8.0 | 2.8 | n/a |
| Chinstrap | Chick | Down | 2014-02-10 | -22.2 | 8.3 | 3.1 | n/a |
| Chinstrap | Chick | Down | 2014-02-10 | -22.1 | 8.0 | 3.1 | n/a |
| Chinstrap | Chick | Down | 2014-02-10 | -21.8 | 7.9 | 3.1 | n/a |
| Chinstrap | Chick | Down | 2014-02-10 | -22.1 | 8.1 | 3.1 | n/a |
| Chinstrap | Chick | Down | 2014-02-10 | -21.9 | 8.0 | 2.8 | n/a |
| Chinstrap | Chick | Down | 2014-02-10 | -22.2 | 7.7 | 3.1 | n/a |
| Chinstrap | Chick | Down | 2014-02-10 | -22.3 | 8.2 | 3.1 | n/a |
| Chinstrap | Chick | Down | 2014-02-10 | -21.5 | 8.2 | 3.1 | n/a |
| Chinstrap | Chick | Down | 2014-02-10 | -21.8 | 8.0 | 3.1 | n/a |
| Chinstrap | Chick | Down | 2014-02-10 | -22.1 | 8.1 | 3.1 | n/a |
| Chinstrap | Chick | Feather | 2014-02-10 | -22.1 | 8.6 | 3.1 | n/a |
| Chinstrap | Chick | Feather | 2014-02-10 | -22.1 | 8.2 | 3.1 | n/a |
| Chinstrap | Chick | Feather | 2014-02-10 | -22.2 | 8.6 | 3.0 | n/a |
| Chinstrap | Chick | Feather | 2014-02-10 | -22.0 | 8.5 | 3.0 | n/a |
| Chinstrap | Chick | Feather | 2014-02-10 | -21.8 | 8.5 | 3.1 | n/a |
| Chinstrap | Chick | Feather | 2014-02-10 | -22.2 | 8.6 | 3.1 | n/a |
| Chinstrap | Chick | Feather | 2014-02-10 | -21.9 | 8.6 | 3.1 | n/a |
| Chinstrap | Chick | Feather | 2014-02-10 | -22.1 | 8.7 | 3.1 | n/a |
| Chinstrap | Chick | Feather | 2014-02-10 | -22.2 | 8.6 | 3.4 | n/a |
| Chinstrap | Chick | Feather | 2014-02-10 | -21.7 | 8.5 | 3.1 | n/a |
| Chinstrap | Chick | Feather | 2014-02-10 | -22.0 | 8.5 | 3.1 | n/a |
| Chinstrap | Chick | Feather | 2014-02-10 | -21.7 | 8.6 | 3.1 | n/a |
| Chinstrap | Chick | Feather | 2014-02-10 | -22.0 | 8.5 | 3.2 | n/a |
| Chinstrap | Chick | Feather | 2014-02-10 | -21.7 | 8.5 | 3.1 | n/a |
| Chinstrap | Chick | Feather | 2014-02-10 | -21.6 | 8.2 | 3.1 | n/a |
| Chinstrap | Chick | Feather | 2014-02-10 | -22.5 | 8.6 | 3.1 | n/a |
| Chinstrap | Chick | Feather | 2014-02-10 | -22.3 | 8.3 | 3.1 | n/a |
| Chinstrap | Chick | Feather | 2014-02-10 | -21.9 | 8.5 | 3.1 | n/a |
| Chinstrap | Chick | Feather | 2014-02-10 | -21.9 | 8.6 | 3.1 | n/a |
| Chinstrap | Chick | Feather | 2014-02-10 | -21.5 | 8.7 | 3.1 | n/a |
| Chinstrap | Chick | Feather | 2014-02-10 | -21.7 | 8.4 | 3.1 | n/a |
| Chinstrap | Chick | Feather | 2014-02-10 | -21.9 | 8.4 | 3.1 | n/a |
| Chinstrap | Chick | Feather | 2014-02-10 | -21.8 | 8.4 | 3.1 | n/a |
| Chinstrap | Chick | Feather | 2014-02-10 | -22.0 | 8.7 | 3.1 | n/a |
| Chinstrap | Chick | Feather | 2014-02-10 | -22.1 | 8.4 | 3.1 | n/a |
| Gentoo | Adult | Blood whole | 2014-02-10 | -22.9 | 8.5 | 3.1 | n/a |
| Gentoo | Adult | Blood whole | 2014-02-10 | -22.9 | 7.9 | 3.1 | n/a |
| Gentoo | Adult | Blood whole | 2014-02-10 | -22.8 | 8.7 | 3.1 | n/a |
| Gentoo | Adult | Blood whole | 2014-02-10 | -22.8 | 8.8 | 3.1 | n/a |
| Gentoo | Adult | Blood whole | 2014-02-10 | -23.1 | 8.0 | 3.1 | n/a |
| Gentoo | Adult | Blood whole | 2014-02-10 | -22.8 | 8.2 | 3.1 | n/a |
| Gentoo | Adult | Blood whole | 2014-02-10 | -23.1 | 8.5 | 3.1 | n/a |
| Gentoo | Adult | Blood whole | 2014-02-10 | -23.1 | 8.1 | 3.1 | n/a |
| Gentoo | Adult | Blood whole | 2014-02-10 | -22.9 | 7.5 | 3.1 | n/a |
| Gentoo | Adult | Blood whole | 2014-02-10 | -23.0 | 8.0 | 3.1 | n/a |
| Gentoo | Adult | Blood whole | 2014-02-10 | -23.0 | 8.1 | 3.1 | n/a |
| Gentoo | Adult | Blood whole | 2014-02-10 | -22.8 | 8.1 | 3.1 | n/a |
| Gentoo | Adult | Blood whole | 2014-02-10 | -23.1 | 7.7 | 3.2 | n/a |
| Gentoo | Adult | Blood whole | 2014-02-10 | -22.6 | 8.7 | 3.3 | n/a |
| Gentoo | Adult | Blood whole | 2014-02-10 | -22.9 | 8.1 | 3.3 | n/a |
| Gentoo | Adult | Plasma | 2015-12-23 | -24.6 | 9.5 | 3.6 | -24.0 |
| Gentoo | Adult | Plasma | 2015-12-23 | -25.1 | 9.4 | 3.8 | -24.1 |
| Gentoo | Adult | Plasma | 2015-12-23 | -25.3 | 8.4 | 3.6 | -24.5 |
| Gentoo | Adult | Plasma | 2015-12-31 | -25.6 | 9.1 | 3.9 | -24.6 |
| Gentoo | Adult | Plasma | 2015-12-31 | -25.3 | 8.5 | 3.6 | -24.6 |
| Gentoo | Adult | Plasma | 2015-12-31 | -25.3 | 8.5 | 3.6 | -24.6 |
| Gentoo | Adult | Plasma | 2016-01-28 | -24.4 | 8.8 | 3.6 | -23.6 |
| Gentoo | Adult | Plasma | 2016-01-28 | -24.6 | 8.5 | 3.7 | -23.8 |
| Gentoo | Adult | Plasma | 2016-01-28 | -25.4 | 8.5 | 4.2 | -24.1 |
| Gentoo | Adult | Plasma | 2016-01-28 | -25.7 | 8.3 | 4.3 | -24.2 |
| Gentoo | Adult | Plasma | 2016-02-09 | -24.5 | 8.2 | 3.8 | -23.6 |
| Gentoo | Adult | Plasma | 2016-02-09 | -25.8 | 9.4 | 5.4 | -23.8 |
| Gentoo | Adult | Plasma | 2016-02-09 | -25.6 | 9.1 | 5.0 | -23.8 |
| Gentoo | Adult | Plasma | 2016-02-09 | -26.4 | 8.7 | 6.0 | -24.2 |
| Gentoo | Adult | Plasma | 2016-02-09 | -27.1 | 8.4 | 8.0 | -24.5 |
| Gentoo | Adult | Red blood cells | 2015-12-23 | -24.1 | 9.3 | 3.2 | n/a |
| Gentoo | Adult | Red blood cells | 2015-12-23 | -24.6 | 8.3 | 3.3 | n/a |
| Gentoo | Adult | Red blood cells | 2015-12-23 | -24.4 | 8.8 | 3.3 | n/a |
| Gentoo | Adult | Red blood cells | 2015-12-23 | -24.1 | 9.2 | 3.3 | n/a |
| Gentoo | Adult | Red blood cells | 2015-12-23 | -24.5 | 8.1 | 3.2 | n/a |
| Gentoo | Adult | Red blood cells | 2015-12-24 | -24.5 | 8.0 | 3.2 | n/a |
| Gentoo | Adult | Red blood cells | 2015-12-31 | -24.7 | 8.0 | 3.3 | n/a |
| Gentoo | Adult | Red blood cells | 2015-12-31 | -24.6 | 7.8 | 3.2 | n/a |
| Gentoo | Adult | Red blood cells | 2015-12-31 | -24.6 | 8.3 | 3.2 | n/a |
| Gentoo | Adult | Red blood cells | 2015-12-31 | -24.8 | 8.1 | 3.3 | n/a |
| Gentoo | Adult | Red blood cells | 2016-01-01 | -24.5 | 8.6 | 3.3 | n/a |
| Gentoo | Adult | Red blood cells | 2016-01-01 | -24.3 | 9.1 | 3.3 | n/a |
| Gentoo | Adult | Red blood cells | 2016-01-28 | -24.9 | 8.3 | 3.4 | n/a |
| Gentoo | Adult | Red blood cells | 2016-01-28 | -24.7 | 7.8 | 3.2 | n/a |
| Gentoo | Adult | Red blood cells | 2016-01-28 | -24.7 | 8.1 | 3.2 | n/a |
| Gentoo | Adult | Red blood cells | 2016-01-28 | -24.8 | 7.7 | 3.2 | n/a |
| Gentoo | Adult | Red blood cells | 2016-01-28 | -24.6 | 7.8 | 3.2 | n/a |
| Gentoo | Adult | Red blood cells | 2016-02-09 | -24.9 | 7.3 | 3.2 | n/a |
| Gentoo | Adult | Red blood cells | 2016-02-09 | -24.5 | 9.3 | 3.5 | n/a |
| Gentoo | Adult | Red blood cells | 2016-02-09 | -24.3 | 8.8 | 3.4 | n/a |
| Gentoo | Adult | Red blood cells | 2016-02-09 | -24.5 | 8.8 | 3.5 | n/a |
| Gentoo | Adult | Red blood cells | 2016-02-09 | -24.6 | 8.0 | 3.5 | n/a |
| Gentoo | Chick | Down | 2014-02-10 | -22.1 | 8.5 | 3.1 | n/a |
| Gentoo | Chick | Down | 2014-02-10 | -22.3 | 9.0 | 3.1 | n/a |
| Gentoo | Chick | Down | 2014-02-10 | -22.3 | 8.7 | 3.1 | n/a |
| Gentoo | Chick | Down | 2014-02-10 | -22.3 | 9.4 | 3.1 | n/a |
| Gentoo | Chick | Down | 2014-02-10 | -21.9 | 8.5 | 3.1 | n/a |
| Gentoo | Chick | Down | 2014-02-10 | -22.4 | 8.8 | 3.1 | n/a |
| Gentoo | Chick | Down | 2014-02-10 | -21.4 | 10.3 | 3.1 | n/a |
| Gentoo | Chick | Down | 2014-02-10 | -22.5 | 8.5 | 3.1 | n/a |
| Gentoo | Chick | Down | 2014-02-10 | -22.1 | 8.4 | 3.1 | n/a |
| Gentoo | Chick | Down | 2014-02-10 | -22.0 | 8.8 | 3.1 | n/a |
| Gentoo | Chick | Down | 2014-02-10 | -22.2 | 8.8 | 3.1 | n/a |
| Gentoo | Chick | Down | 2014-02-10 | -22.1 | 8.7 | 3.1 | n/a |
| Gentoo | Chick | Down | 2014-02-10 | -22.2 | 8.5 | 3.1 | n/a |
| Gentoo | Chick | Down | 2014-02-10 | -22.2 | 8.2 | 3.1 | n/a |
| Gentoo | Chick | Down | 2014-02-10 | -22.2 | 9.3 | 3.1 | n/a |
| Gentoo | Chick | Down | 2014-02-10 | -22.1 | 8.6 | 3.1 | n/a |
| Gentoo | Chick | Down | 2014-02-10 | -22.2 | 8.5 | 3.1 | n/a |
| Gentoo | Chick | Down | 2014-02-10 | -22.1 | 9.0 | 3.0 | n/a |
| Gentoo | Chick | Down | 2014-02-10 | -22.0 | 8.6 | 3.1 | n/a |
| Gentoo | Chick | Down | 2014-02-10 | -22.2 | 8.5 | 3.1 | n/a |
| Gentoo | Chick | Down | 2014-02-10 | -21.6 | 9.3 | 3.1 | n/a |
| Gentoo | Chick | Down | 2014-02-10 | -22.0 | 8.6 | 3.1 | n/a |
| Gentoo | Chick | Down | 2014-02-10 | -21.5 | 9.9 | 3.1 | n/a |
| Gentoo | Chick | Down | 2014-02-10 | -21.6 | 8.5 | 3.1 | n/a |
| Gentoo | Chick | Down | 2016-02-03 | -24.1 | 8.5 | 3.0 | n/a |
| Gentoo | Chick | Down | 2016-02-03 | -23.1 | 8.8 | 3.0 | n/a |
| Gentoo | Chick | Down | 2016-02-03 | -21.6 | 11.8 | 3.0 | n/a |
| Gentoo | Chick | Down | 2016-02-03 | -23.5 | 9.5 | 3.0 | n/a |
| Gentoo | Chick | Down | 2016-02-03 | -23.9 | 8.9 | 3.0 | n/a |
| Gentoo | Chick | Down | 2016-02-03 | -23.5 | 9.4 | 3.1 | n/a |
| Gentoo | Chick | Down | 2016-02-03 | -24.0 | 8.2 | 3.1 | n/a |
| Gentoo | Chick | Down | 2016-02-03 | -23.3 | 9.2 | 3.1 | n/a |
| Gentoo | Chick | Down | 2016-02-03 | -23.0 | 9.4 | 3.1 | n/a |
| Gentoo | Chick | Down | 2016-02-03 | -23.4 | 9.0 | 3.1 | n/a |
| Gentoo | Chick | Feather | 2014-02-10 | -21.4 | 10.1 | 3.1 | n/a |
| Gentoo | Chick | Feather | 2014-02-10 | -21.3 | 9.9 | 3.1 | n/a |
| Gentoo | Chick | Feather | 2014-02-10 | -21.2 | 10.2 | 3.1 | n/a |
| Gentoo | Chick | Feather | 2014-02-10 | -20.5 | 10.4 | 3.1 | n/a |
| Gentoo | Chick | Feather | 2014-02-10 | -21.2 | 9.2 | 3.1 | n/a |
| Gentoo | Chick | Feather | 2014-02-10 | -21.4 | 9.3 | 3.2 | n/a |
| Gentoo | Chick | Feather | 2014-02-10 | -20.6 | 10.7 | 3.1 | n/a |
| Gentoo | Chick | Feather | 2014-02-10 | -21.3 | 9.2 | 3.1 | n/a |
| Gentoo | Chick | Feather | 2014-02-10 | -21.2 | 8.8 | 3.1 | n/a |
| Gentoo | Chick | Feather | 2014-02-10 | -21.0 | 10.4 | 3.1 | n/a |
| Gentoo | Chick | Feather | 2014-02-10 | -21.3 | 9.5 | 3.1 | n/a |
| Gentoo | Chick | Feather | 2014-02-10 | -21.2 | 9.0 | 3.1 | n/a |
| Gentoo | Chick | Feather | 2014-02-10 | -20.7 | 10.7 | 3.1 | n/a |
| Gentoo | Chick | Feather | 2014-02-10 | -21.4 | 8.7 | 3.1 | n/a |
| Gentoo | Chick | Feather | 2014-02-10 | -20.6 | 9.8 | 3.1 | n/a |
| Gentoo | Chick | Feather | 2014-02-10 | -21.3 | 9.5 | 3.1 | n/a |
| Gentoo | Chick | Feather | 2014-02-10 | -21.9 | 9.3 | 3.1 | n/a |
| Gentoo | Chick | Feather | 2014-02-10 | -21.3 | 10.3 | 3.1 | n/a |
| Gentoo | Chick | Feather | 2014-02-10 | -21.0 | 9.2 | 3.1 | n/a |
| Gentoo | Chick | Feather | 2014-02-10 | -20.4 | 10.1 | 3.1 | n/a |
| Gentoo | Chick | Feather | 2014-02-10 | -21.2 | 9.1 | 3.1 | n/a |
| Gentoo | Chick | Feather | 2014-02-10 | -20.7 | 11.0 | 3.1 | n/a |
| Gentoo | Chick | Feather | 2014-02-10 | -21.1 | 9.5 | 3.1 | n/a |
| Gentoo | Chick | Feather | 2014-02-10 | -20.9 | 9.9 | 3.1 | n/a |
| Gentoo | Chick | Feather | 2016-02-03 | -22.4 | 9.7 | 3.1 | n/a |
| Gentoo | Chick | Feather | 2016-02-03 | -22.9 | 8.7 | 3.1 | n/a |
| Gentoo | Chick | Feather | 2016-02-03 | -20.9 | 12.3 | 3.1 | n/a |
| Gentoo | Chick | Feather | 2016-02-03 | -22.8 | 9.9 | 3.1 | n/a |
| Gentoo | Chick | Feather | 2016-02-03 | -22.9 | 9.4 | 3.2 | n/a |
| Gentoo | Chick | Feather | 2016-02-03 | -22.8 | 9.3 | 3.1 | n/a |
| Gentoo | Chick | Feather | 2016-02-03 | -22.7 | 10.4 | 3.1 | n/a |
| Gentoo | Chick | Feather | 2016-02-03 | -22.8 | 9.4 | 3.1 | n/a |
| Gentoo | Chick | Feather | 2016-02-03 | -22.5 | 9.6 | 3.1 | n/a |

**Table S3.** Carbon and nitrogen isotopic ratios measured in 366 samples from three pygoscelid penguin species from Powell Island, South Orkney Islands. Corrected *δ*^13^C values were obtained through normalization equations applied solely to plasma samples (see methods for details). For all other tissues, *δ*^13^C bulk values were used. Finally, bulk *δ*^15^N values were used for all tissues, including plasma (see methods for details).
